# *Geobacillus* Bacteriophages from Compost Heaps: Representatives of Three New Genera within Thermophilic Siphoviruses

**DOI:** 10.3390/v15081691

**Published:** 2023-08-04

**Authors:** Eugenijus Šimoliūnas, Monika Šimoliūnienė, Gintarė Laskevičiūtė, Kotryna Kvederavičiūtė, Martynas Skapas, Algirdas Kaupinis, Mindaugas Valius, Rolandas Meškys, Nomeda Kuisienė

**Affiliations:** 1 Department of Molecular Microbiology and Biotechnology, Institute of Biochemistry, Life Sciences Center, Vilnius University, Saulėtekio Av. 7, LT-10257 Vilnius, Lithuania; monika.simoliuniene@gmc.vu.lt (M.Š.); gintare.laskeviciute@gmail.com (G.L.); rolandas.meskys@bchi.vu.lt (R.M.); 2 Department of Microbiology and Biotechnology, Institute of Bioscience, Life Sciences Center, Vilnius University, Saulėtekio Av. 7, LT-10257 Vilnius, Lithuania; nomeda.kuisiene@gf.vu.lt; 3 Department of Biological DNA Modification, Institute of Biotechnology, Life Sciences Center, Vilnius University, Saulėtekio Av. 7, LT-10257 Vilnius, Lithuania; kotryna.kvederaviciute@mif.vu.lt; 4 Department of Characterisation of Materials Structure, Center for Physical Sciences and Technology, Sauletekio Av. 3, LT-10257 Vilnius, Lithuania; martynas.skapas@ftmc.lt; 5 Proteomics Centre, Institute of Biochemistry, Life Sciences Center, Vilnius University, Saulėtekio Av. 7, LT-10257 Vilnius, Lithuania; algirdas.kaupinis@gf.vu.lt (A.K.); mindaugas.valius@bchi.vu.lt (M.V.)

**Keywords:** phages, thermophiles, viruses, depolymerases, genomic analysis

## Abstract

We report a detailed characterization of five thermophilic bacteriophages (phages) that were isolated from compost heaps in Vilnius, Lithuania using *Geobacillus thermodenitrificans* strains as the hosts for phage propagation. The efficiency of plating experiments revealed that phages formed plaques from 45 to 80 °C. Furthermore, most of the phages formed plaques surrounded by halo zones, indicating the presence of phage-encoded bacterial exopolysaccharide (EPS)-degrading depolymerases. Transmission Electron Microscopy (TEM) analysis revealed that all phages were siphoviruses characterized by an isometric head (from ~63 nm to ~67 nm in diameter) and a non-contractile flexible tail (from ~137 nm to ~150 nm in length). The genome sequencing resulted in genomes ranging from 38,161 to 39,016 bp. Comparative genomic and phylogenetic analysis revealed that all the isolated phages had no close relatives to date, and potentially represent three new genera within siphoviruses. The results of this study not only improve our knowledge about poorly explored thermophilic bacteriophages but also give new insights for further investigation of thermophilic and/or thermostable enzymes of bacterial viruses.

## 1. Introduction

*Geobacillus* is a genus of thermophilic, endospore-forming, chemo-organotrophic, and rod-shaped Gram-positive bacteria of the family *Bacillaceae* [1,2]. Members of this genus are widespread and have been isolated not only from extreme environments such as hot springs, high-temperature oil fields, hydrothermal vents, composts, and greenhouse soil, but have also been extensively found in cold places, much below their minimal growth temperatures, such as soil samples or ocean sediments [2,3]. *Geobacillus* spp. are broadly exploited in various biotechnological and industrial applications [4,5], and promising results about the antimicrobial potential of these bacteria have been reported [6]. In contrast, the role of the biotic factors, and especially of bacteriophages (phages) affecting *Geobacillus* spp., remains poorly explored.

A limited number of *Geobacillus* bacteriophages with completely sequenced genomes have been published to date including phages GVE2 (E2) [7], GVE3 [8], D6E [9], GBK2 [10], GBSV1 [11], BV1 [12], φOH2 [13], TP-84 [14], and GR1 (genome of this phage is deposited in the NCBI database, but has not yet been released to date, accessed on 11 July 2023) [15]. In addition, *Geobacillus* virus GVE1 has been characterized [16], but the complete genome of this phage has not been published to date (accessed in the NCBI database on 11 July 2023). In addition, a number of bacteriophages infecting *Bacillus stearothermophilus* strains (according to current classification—*G. stearothermophilus*) have been reported in the past century, although no complete genomes of these phages have been published to date [17]. Finally, genomes of two *Geobacillus* phages, which are vB_GthS_PK5.2 (OP341629.1) and vB_GthS_PK2.1 (OP341625.1), have been deposited in the NCBI database by our research group but not characterized to date.

In this study, we present biological characteristics and complete genome analysis of five *Geobacillus thermodenitrificans*-infecting siphoviruses: vB_GthS_PT9.1, vB_GthS_NIIg9.7, vB_GthS_PK5.1, vB_GthS_PK3.5, and vB_GthS_PK3.6, referred here by their shorter names PT9.1, NIIg9.7, PK5.1, PK3.5 and PK3.6, respectively. All phages show a high-temperature plating profile. Moreover, phages PT9.1, PK5.1, and PK3.6 demonstrate an ability to form plaques even at 80 °C. The phylogenetic analysis indicates that the isolated bacteriophages are phylogenetically the most closely interconnected to each other, but distant from already known viruses, and likely represent three new genera within the siphophages. Thus, the data presented here not only provide information on the morphology, physiology, and genetic diversity of *Geobacillus*-infecting viruses, but also broaden our understanding of virus–host interactions in dynamic ecosystems, such as compost heaps.

## 2. Materials and Methods

### 2.1. Phages and Bacterial Strains

Bacteriophages and bacterial strains were originally isolated from soil samples collected from compost heaps at Vilnius University Botanical Garden, Vingis Park, Vilnius, Lithuania (54.682912, 25.232532). Five *Geobacillus* bacteriophages are described in this study, namely vB_GthS_PT9.1, vB_GthS_NIIg9.7, vB_GthS_PK5.1, vB_GthS_PK3.5, and vB_GthS_PK3.6. The bacterial strains used in this study are listed in Table S1. Bacterial strains were isolated by using deep agar dilution series. For all of the phage experiments, the bacterial strains were cultivated in Luria–Bertani (LB) broth (Formedium). Solid plates were prepared by adding the appropriate amount of agar (Formedium) or gellan (PanReac Applichem) to the liquid medium. To identify isolated bacterial strains, PCR amplification of 16S rRNA gene fragment was performed by using universal primers woo1 5′-AGAGTTTGATCMTGGCTC-3′ and woo2 5′-GNTACCTTGTTACGACTT-3′ [18]. The BOX element was amplified using the BOXA1R primer 5′-CTACGGCAAGGCGACGCTGACG-3′ [19]. The PCR reaction for each isolate was repeated thrice for reproducibility.

### 2.2. Phage Isolation, Propagation, and Purification Techniques

Phage isolation was performed by using the technique of enrichment of phages in the source material. Briefly, soil samples of compost heaps (5–10 g) were filled up with 10 mL of LB and incubated for one week at 50 °C, followed by low-speed centrifugation at 2800× *g* for 15 min. The supernatant was then sequentially filtered through sterile 0.45 and 0.2 µm membrane filters and was assayed for plaque-forming units using the soft agar overlay method described by Adams [20], with minor modifications. Concisely, 0.1 mL of diluted phage suspension was mixed with 0.5 mL of indicator cells (OD_600_–0.5). The mixture then was added to 3 mL of 0.4% (*w/v*) soft agar and poured over the 1.2% (*w/v*) LB agar plate as a uniform layer. The plates were incubated for 24 h at 55 °C before the enumeration of plaques. The efficiency of plating experiments was made at 40–85 °C, and incubation of plates above 75 °C was performed by using 3 mL of 0.1% (*w*/*v*) soft gellan and poured over the 0.75% (*w/v*) gellan plate as a uniform layer. Bacteriophages were purified by performing five consecutive transfers from individual plaques to new bacterial cell lawns. Notably, as isolated thermophilic bacterial strains were growing poorly in the liquid broth, the propagation of bacteriophages was performed using the soft agar overlay method, as described previously by Šimoliūnas et al. [21], with minor modifications. Briefly, phage particles were subsequently collected by adding 5 mL of PB buffer (70 mM NaCl, 10 mM MgSO_4_, 50 mM Na_2_HPO_4_, 30 mM KH_2_PO_4_) to the surface of each plate. The top agar was scraped off and the suspension recovered. After 30 min of incubation at 4 °C with mild stirring, the mixture was centrifuged at 2800× *g* for 15 min at 4 °C. The phage-containing supernatant was collected and filtered through sterile 0.45 µm membrane filters. Phages were concentrated by high-speed centrifugation at 16,000× *g* for 1 h at 4 °C. The resulting pellets were suspended in PB buffer. To avoid bacterial DNA contamination, DNase I was added to the phage suspensions, and the samples were incubated for 1 h at 37 °C. Further phage purification was performed using a CsCl step gradient [22] as described by Šimoliūnas et al. [23].

### 2.3. Transmission Electron Microscopy

The CsCl density gradient-purified phage particles were diluted to approximately 10^10^–10^11^ PFU/mL with distilled water, and 10 µL of the sample was directly applied onto the carbon-coated nickel grid (Agar Scientific, Essex, UK). After 1 min, the excess liquid was drained with filter paper and stained with two successive drops of 2% uranyl acetate (pH 4.5) for 1 min. The sample was then dried and examined using a Tecnai G2 F20 X-TWIN transmission electron microscope (FEI, Hillsboro, OR, USA).

### 2.4. DNA Isolation

The aliquots of high-titer (10^11^–10^12^ PFU/mL) phage suspensions were subjected to phenol/chloroform extraction and ethanol precipitation, as described by Carlson and Miller [24]. The isolated phage DNA was subsequently used for PCR, or it was subjected to genome sequencing.

### 2.5. Genome Sequencing and Analysis

The complete genome sequences of the bacteriophages were determined using Illumina DNA sequencing technology at Macrogen (Amsterdam, The Netherlands). DNA libraries were prepared using TruSeq DNA PCR Free (350) library preparation. The 151 bp length paired-end sequence reads were generated using the NovaSeq (6000) platform.

FASTQ read sequence files were generated using bcl2fastq version 2.20 (Illumina, San Diego, CA, USA). The quality of the raw reads was evaluated using FASTQC quality control tool version 0.11.9 [25] (available online: http://www.bioinformatics.babraham.ac.uk/projects/fastqc/, accessed on 11 July 2023). Low-quality bases and adapters were trimmed using TrimGalore version 0.6.6 [26] (available online: https://www.bioinformatics.babraham.ac.uk/projects/trim_galore/, accessed on 11 July 2023) using standard parameters. Samples were downsampled using reformat.sh tool from BBMAP package version 38.96 (available online: https://sourceforge.net/projects/bbmap/files/, accessed on 11 July 2023) up to approx. 70M reads each. The quality of the reads was improved using BayesHammer [27] bundled with the SPAdes package and genomes were assembled using SPAdes packages version 3.13.1 [28]. The BBMAP package (v38.96) was used to evaluate the mapping rate and coverage. The reads of the bacteriophages were assembled into single linear contigs ranging from 38,238 (phage PK5.1) to 39,093 (phage NIIg9.7) bp with an average coverage from 537.144 (phage NIIg9.7) to 546.829 (phage PT9.1) (Table S2). The ends of the contigs were confirmed using PCR, followed by Sanger sequencing reactions at Macrogen (Amsterdam, The Netherlands). PCR fragments were obtained by the amplification of phage wild-type DNA using the primers presented in Table S2. PhageTerm [29] was used for the determination of phage termini. No known packaging mechanisms were identified in any of the genomes, and to preserve gene contiguity, the genome start points were selected from the predicted terminase small subunit gene.

The open reading frames (ORFs) were predicted with Geneious Prime version 2023.1 (available online: http://www.geneious.com/, accessed on 11 July 2023) using a minimum ORF size of 60 nt. The analysis of the genome sequences was performed using BLASTp, Fasta-Protein, Fasta-Nucleotide, Transeq (available online: http://www.ebi.ac.uk/Tools/st/emboss_transeq/, accessed on 11 July 2023), Clustal Omega (available online: http://www.ebi.ac.uk/Tools/msa/clustalo/, accessed on 11 July 2023), and DNA sequence editor available online: http://www.biocourseware.com/iphone/dnaseqeditor/index.htm/, accessed on 11 July 2023), as well as HHPred and HHblits [30,31]. The tRNAscan-SE 2.0 (available online: http://lowelab.ucsc.edu/tRNAscan-SE/, accessed on 11 July 2023) and ARAGORN (available online: http://www.ansikte.se/ARAGORN/, accessed on 11 July 2023) were used to search for tRNAs. Neighbor-joining phylogenetic tree analysis was conducted using MEGA version 5 [32]. ViPTree [33] version 3.6 was used for the total proteome comparisons (available online: https://www.genome.jp/viptree/, accessed on 15 June 2023). The overall nucleotide sequence identity was calculated using VIRIDIC (intergenomic distance calculator) [34].

### 2.6. Analysis of Structural Proteins

An analysis of the structural proteins of phage virions was performed using a modified filter-aided sample preparation (FASP) protocol, followed by Liquid Chromatography with Tandem Mass Spectrometry (LC-MS/MS) analysis, as described by Šimoliūnas et al. [21].

### 2.7. Nucleotide Sequence Accession Numbers

The complete genome sequences of *Geobacillus* bacteriophages were deposited in the EMBL nucleotide sequence database under the following accession numbers: vB_GthS_PT9.1 (OP341630), vB_GthS_NIIg9.7 (OP341624), vB_GthS_PK5.1 (OP341628), vB_GthS_PK3.5 (OP341626), and vB_GthS_PK3.6 (OP341627). The accession numbers of the PCR-amplified 16S rRNA gene sequences of *Bacillus*-group bacteria isolated during this study are presented in Table S1.

## 3. Results

### 3.1. Identification and Characterization of the Bacterial Isolates

**Figure 1:**
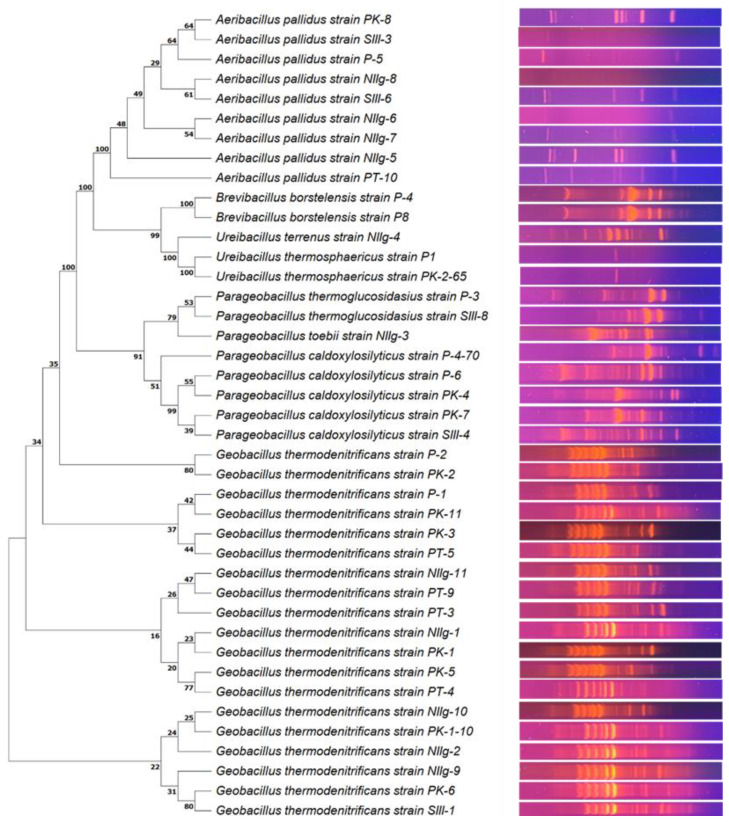
Neighbor-joining phylogenetic tree analysis based on the alignment of the 16S rRNA gene fragment nucleotide sequences (**left**) and the BOX-PCR electrophoresis pattern (**right**) of isolated thermophilic bacterial strains. Neighbor-joining bootstrap values are indicated at each branch.

In total, 41 different bacterial strains were isolated during this study based on the results of morphological, physiological, and genetic analysis (Table S1). Genetic analysis was based on the comparison of PCR-amplified 16S rRNA gene fragment sequences. In addition, BOX-PCR, which can be applied for molecular typing within the *Geobacillus* genus [35], was performed (Figure 1). It was demonstrated that all isolated thermophilic bacteria are the members of phylogenetically closely related genera: *Geobacillus* (nineteen strains), *Aeribacillus* (nine strains), *Parageobacillus* (eight strains), *Ureibacillus* (three strains) and *Brevibacillus* (two strains).

Isolated bacteria formed colonies of various morphology (Figure S1) and demonstrated different growth temperature ranges (Figure S2). *Brevibacillus borstelensis* strains P8 and P-4 demonstrated the lowest growth temperature (from 22 to 55 °C) whereas six *Geobacillus thermodenitrificans* strains (NIIg-1, NIIg-2, NIIg-9, NIIg-11, PK-1-10, and PT-4) propagated even at 80 °C under investigated conditions. In addition, all tested strains were able to grow on LB agar/gellan medium but demonstrate limited growth in liquid LB medium. Isolated bacterial strains were used as hosts for phage isolation and propagation experiments. Bacteriophages were recovered from the same compost samples from which the bacteria were isolated.

### 3.2. Host Range, Morphology, and Physiological Characteristics of the Phages

Bacteriophages PT9.1, NIIg9.7, PK5.1, PK3.5, and PK3.6 were isolated by using the technique of enrichment of phages in the source material as previously described (Section 2.2), using the local isolates PT9, NIIg9, PK5, and PK3 of *Geobacillus thermodenitrificans*, accordingly, as hosts. In further experiments, 46 thermophilic bacterial strains closely related by clades were used to explore the host range of the isolated *Geobacillus* bacteriophages (Table S3). With the exception of *Geobacillus thermodenitrificans* strains, the other tested *Geobacillus* spp., as well as all of the tested strains of *Aeribacillus*, *Brevibacillus*, *Parageobacillus*, *Peribacillus*, and *Ureibacillus* spp., were found to be resistant to the isolated phages. However, phages demonstrated different host ranges: NIIg9.7 and PT9.1 were active against nine, PK3.5 lysed four, whereas PK3.6 and PK5.1 infected only two of the tested strains.

**Figure 2:**
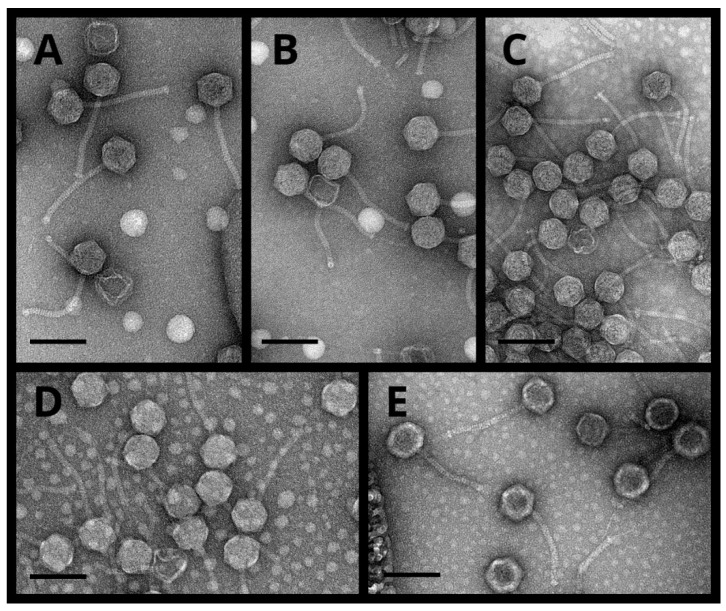
Electron micrographs of bacteriophages PT9.1 (**A**), NIIg9.7 (**B**), PK5.1 (**C**), PK3.5 (**D**), and PK3.6 (**E**). Scale bar represents 100 nm.

Transmission electron microscopy observations of the phages PT9.1, NIIg9.7, PK5.1, PK3.5, and PK3.6 (Figure 2) revealed particles that all fit the B1 morphotype in Bradley’s classification [36,37]. Based on the morphological characteristics, all five phages were siphoviruses characterized by isometric heads and non-contractile tails (Figure 2). The phages PT9.1 and PK5.1 possessed the smallest heads (diameter of 62.72 ± 2.34 nm and 62.91 ± 3.20 nm, respectively), whereas the phage PK3.6 was the largest of the viruses examined in this study, with a head diameter of 66.93 ± 4.24 nm. Phages PK3.5 and PK3.6 contained the longest (149.98 ± 15.01 nm) and the shortest (137.32 ± 5.75 nm) tails, accordingly. Notably, no short or long tail fibers were clearly visible using TEM. The morphological features of bacteriophages described in this study are presented in Table 1.

**Figure 3:**
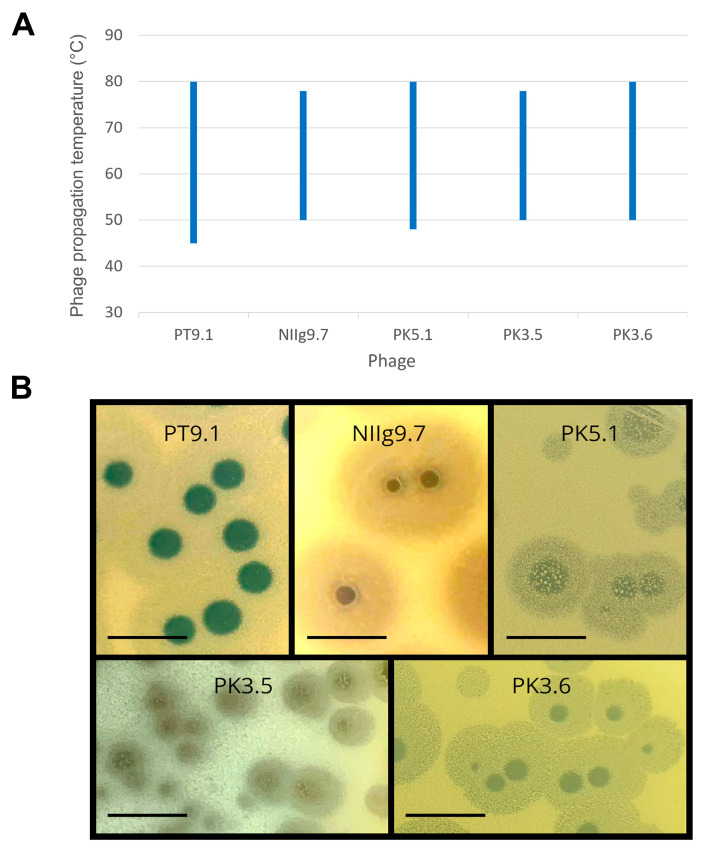
Propagation temperature range (**A**) and morphology of plaques (**B**) of the thermophilic bacteriophages. Determination of phage propagation temperature range was examined by using *Geobacillus thermodenitrificans* strains PT-9 (phage PT9.1), NIIg-9 (phage NIIg9.7), PK-5 (phage PK5.1), and PK-3 (phages PK3.5 and PK3.6) as the hosts. The morphology of the plaque-forming units was monitored after 24 h of incubation at 55 °C by using the same hosts as for phage propagation temperature range experiments. Scale bar represents 1 cm.

To determine the optimal conditions for phage propagation, the effect of temperature on the efficiency of plating (e.o.p.) was examined in the temperature range of 40–85 °C (Figure 3A). It was demonstrated that phages NIIg9.7 and PK3.5 infected their host cells from 50 to 78 °C. Moreover, PT9.1, PK5.1, and PK3.6 formed plaques even at 80 °C, and the lowest temperatures for their infectivity are 45, 48, and 50 °C, accordingly. In addition, all phages formed plaques with a clear center surrounded by an opaque halo zone (Figure 3B). Phage NIIg9.7 formed plaques with the largest halo zones; after one day of incubation at 55 °C, these were up to 11.42 ± 0.75 mm in diameter (Table 1, Figure 3B). As it was mentioned previously, all tested host strains demonstrated limited growth in liquid LB medium; thus, it was not possible to perform the adsorption test and/or single-step experiments under the investigated conditions.

### 3.3. Overview of Viral Genomes

**Figure 4:**
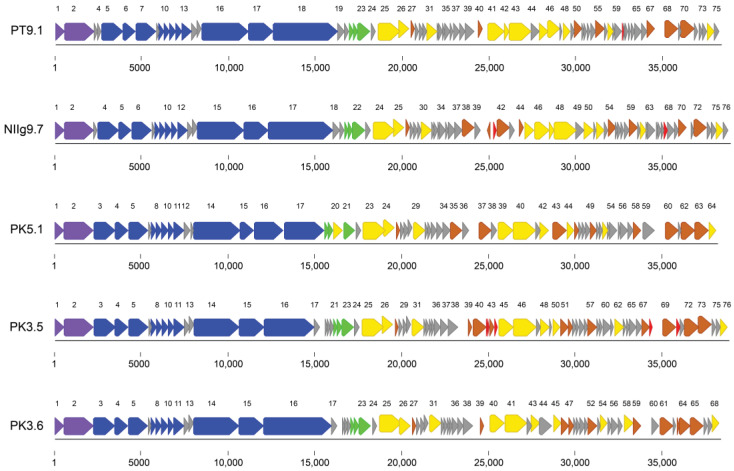
Functional genome maps of *Geobacillus* bacteriophages. The coding capacity of the genomes is shown. Numbers indicate ORF position in genome, and functions are assigned according to the characterized ORFs in the NCBI database and HHpred analysis. The color code is as follows: yellow—DNA replication, recombination, and repair; blue—structural proteins; purple—DNA packaging; brown—transcription, translation, nucleotide metabolism; green—lysis, phage–host interaction; grey—conserved hypothetical proteins; red—hypothetical proteins with no reliable identity when compared to database entries.

The summary of general genomic features of the isolated phages is shown in Table 2. All bacteriophages contained double-stranded DNA genomes varying from 38,161 bp (phage PK5.1) to 39,016 bp (phage NIIg9.7). Phages possessed genomes with a GC content from 43.5% (phage PK3.5) to 44.8% (phage PK3.6), which were insignificantly lower than that (48.8–53.1%) observed for *Geobacillus* species [38]. Similar to other dsDNA bacteriophages, the genomes of thermophilic phages were close packed—92.0% (phage PK3.6) to 94.2% (phage PT9.1) of the genomes were coding. The analysis of the genome sequences revealed that a number of predicted ORFs encoding for proteins ranged from 64 (phage PK5.1) to 76 (phages NIIg9.7 and PK3.5), but no open reading frames encoding for tRNAs were identified (Table 2; Tables S4–S8). Notably, an apparent asymmetry in the distribution of the genes on the two DNA strands of phages was observed. With the exception of ORF40 encoding a ribbon–helix–helix domain-containing protein from phage NIIg9.7, all other ORFs have been predicted to be transcribed from the same DNA strands (Figure 4).

Based on homology to biologically defined proteins, the percentage of ORFs after a putative functional annotation ranges from 45% (34 out of 75 PT9.1 ORFs) to 58% (37 out of 64 PK5.1 ORFs). As was observed in other siphoviruses, the genomes of *Geobacillus* phages appeared to have a modular organization, with genes for DNA packaging, structure/morphogenesis, host lysis, replication/regulation, transcription/translation, and nucleotide metabolism clustered together (Figure 4). Notably, none of the predicted gene products showed sequence homology with antibiotic resistance determinants or integration-related proteins.

### 3.4. Structural Proteins and Proteomic Analysis

A bioinformatics analysis of the genome sequences of isolated bacteriophages allowed for the identification of a number of genes coding for proteins involved in virion structure and assembly (Figure 4; Tables S4–S8). Eleven structural genes, including those coding for the head (portal protein, Clp protease, major capsid protein, head-tail connector, and head closure protein), tail (two putative tail components, major tail protein, tape measure protein, distal tail protein), and tail fiber (tail fiber protein), were identified in the genomes of phages PT9.1, NIIg9.7, PK3.5, and PK3.6. Phage PK5.1 contained twelve structural proteins, including those eleven mentioned above and a structural protein encoded by ORF16, which shares the highest identity (27% amino acid sequence identity; E-value of 3 × 10^–17^) to the putative tail fiber protein (Sequence ID: VEV89172.1) from *Staphylococcus* phage Stab22. The PT9.1 gp18, NIIg9.7 gp17, PK5.1 gp17, PK3.5 gp16, and PK3.6 gp16 were identified as tail fiber proteins. Based on the results of BLASTp analysis, the identity at the aa level of PT9.1 gp18 vs. NIIg9.7 gp17 was 97.57% (query cov. 100%; E-value, 0.0), and PK3.5 gp16, vs. PK3.6 gp16 was 85.31% (query cov. 100%; E-value, 0.0). In contrast, PT9.1 gp18 shared only 33.88% (query cov. 48%; E-value, 1 × 10^−61^) and 34.50% (query cov. 48%; E-value, 4 × 10^−61^) aa identity with PK3.5 gp16 and PK3.6 gp16, respectively. Similarly, NIIg9.7 gp17 was only 33.95% (query cov. 48%; E-value, 1 × 10^−61^) and 34.57% (query cov. 48%; E-value, 1 × 10^−61^) identical to PK3.5 gp16 and PK3.6 gp16, respectively. PK5.1 gp17 shared 79.17% (query cov. 15%; E-value, 3 × 10^−51^) and 78.33% (query cov. 15%; E-value, 4 × 10^−50^) aa identity with PT9.1 gp18 and NIIg9.7 gp17, respectively. Meanwhile, no significant similarity of PK5.1 gp17 to PK3.5 gp16 and PK3.6 gp16 was found.

FASP followed by LC-MS/MS confirmed that a number of the aforementioned structural proteins were present in the virions of thermophilic bacteriophages (Table S9). Three structural proteins were identified in the virions of phages PT9.1 (major capsid protein, tape measure protein, and distal tail protein) and PK3.6 (major capsid protein, tape measure protein, and putative tail fiber protein). Four structural proteins were detected in the virions of phages NIIg9.7 (major capsid protein, tape measure protein, distal tail protein, and tail fiber protein) and PK5.1 (Clp protease, major capsid protein, tape measure protein, and structural protein). Finally, five structural proteins, including major capsid protein (gp05), putative tail component (gp09), tape measure protein (gp14), distal tail protein (gp15), and putative tail fiber protein (gp16), were identified in the virions of PK3.5. Indetermination of potential structural proteins, which were identified by bioinformatics approaches but not detected by proteomics analysis, might be due to the incompatibility of these proteins with sample preparation procedures and/or because of their low abundance in virions.

### 3.5. Packaging 

The packaging machine of tailed bacteriophages usually consists of two essential components: a terminase complex and a portal ring [39]. Most characterized terminases consist of a small subunit (TerS) involved in DNA recognition and a large terminase subunit (TerL) containing the ATPase and the endonuclease activities [40]. The genes associated with DNA packaging of isolated thermophilic bacteriophages include all three aforementioned proteins: the TerS and TerL were encoded by ORF01 and ORF02, respectively, and the portal protein is encoded by ORF03 (phages PK5.1, PK3.5, and PK3.6), ORF04 (phage NIIg9.7), and ORF05 (phage PT9.1). All phages possessed TerS containing conserved Terminase_4 (pfam05119) domain and TerL containing conserved Terminase_1 (pfam03354) domain. Also, all phages had a portal protein with conserved Phage_portal (pfam04860) domain.

### 3.6. DNA Replication, Recombination, and Repair

The bioinformatics analysis revealed that the genes associated with DNA replication, recombination, and repair (DNA RRR) of isolated bacteriophages included those coding for an FtsK/SpoIIIE family protein, a replication/relaxation protein (dnaD), an ERF family protein, a replicative DNA helicase, a single-stranded DNA binding (SSB) protein, and a Holliday junction resolvase. In addition, phages PT9.1 and NIIg9.7 possessed a loader and inhibitor of replicative helicase encoded by ORF42 and ORF47, respectively. Moreover, with the exception of NIIg9.7 and PK3.5, each phage encoded three HNH endonucleases–site-specific DNA endonucleases that promote the lateral transfer of their own coding region and flanking DNA between genomes by a recombination-dependent process termed homing [41]. Phage PK3.5 contained two HNH endonucleases, and phage NIIg9.7 possessed two HNH endonucleases and a putative endonuclease (gp45) containing MTES_1575 (pfam18741) conserved domain. However, the genomes of thermophilic bacteriophages contained no homologs to characterize DNA polymerase genes, suggesting that these phages most likely use DNA polymerase of the host cell.

### 3.7. Transcription, Translation, Nucleotide Metabolism, and DNA Modification

Based on the amino acid sequence similarity, a set of genes encoding products potentially involved in transcription, translation, nucleotide metabolism, and DNA modification were present in the genomes of thermophilic bacteriophages. The phages contained transcriptional regulators belonging to families XRE, ArpU, Rha, and RinA (Tables S4–S8) as well as putative transcriptional regulators encoded by ORF45, ORF53, and ORF47 of phages PK5.1, PK3.5 and PK3.6, respectively. In addition, one antirepressor protein encoded by ORF40 of phage PK3.5 was detected. All phages contain metallophosphoesterase with a conserved COG1407 superfamily domain. With the exception of PK5.1, all phages also encoded dUTP diphosphatase containing dUTPase_2 (pfam08761) conserved domain. On the other hand, phage PK5.1 was the only one containing phosphoadenosine phosphosulfate reductase and nucleoside triphosphate pyrophosphohydrolase encoded by ORF43 and ORF49, respectively. Moreover, thymidylate synthase was encoded by ORF38 and ORF35 of phages NIIg9.7 and PK5.1, respectively. With the exception of PK5.1, all phages possessed DNA N-6-adenine-methyltransferase containing Dam (pfam05869) conserved domain. In addition, DNA methyltransferase containing the N6_N4_Mtase (pfam01555) conserved domain was encoded twice in the genomes of phages PK3.5, PK3.6, and PK5.1, and once in phage PT9.1, but it was not detected in the genome of phage NIIg9.7.

### 3.8. Lysis Cassette

All dsDNA phages accomplish host lysis using a muralytic enzyme (known as an endolysin) and a holin, a small membrane protein that permeabilizes the membrane at a programmed time [42]. The lysis cassette of all isolated thermophilic bacteriophages consisted of a hemolysin containing XhlA (pfam10779) conserved domain, a holin, and an endolysin (N-acetylmuramoyl-L-alanine amidase) containing N-terminal MurNAc-LAA (cd02696) and C-terminal SPOR (pfam05036) conserved domains. With the exception of PK5.1, which encoded an HNH endonuclease (gp20) inserted between holin (gp19) and endolysin (gp21), all three lysis proteins were encoded in canonical order (Figure 4). Hemolysins with the XhlA family motif are cell-surface-associated proteins that lyse insect granulocytes and plasmatocytes, as well as rabbit and horse erythrocytes [43]. However, it was demonstrated that proteins similar to XhlA, which were encoded by the *Bacillus* phage SPP1 and prophage PBSX, functioned as holins [44]. The presence of holin and XhlA domain-containing proteins had also been described in the case of other *Bacillus*-group bacteria infecting phages [7,45,46]. It is likely that activation of more than one holin may facilitate the lysis of cells grown in different conditions or coming from different phage hosts [44].

### 3.9. Phylogenetic Analysis of the Phages

**Figure 5:**
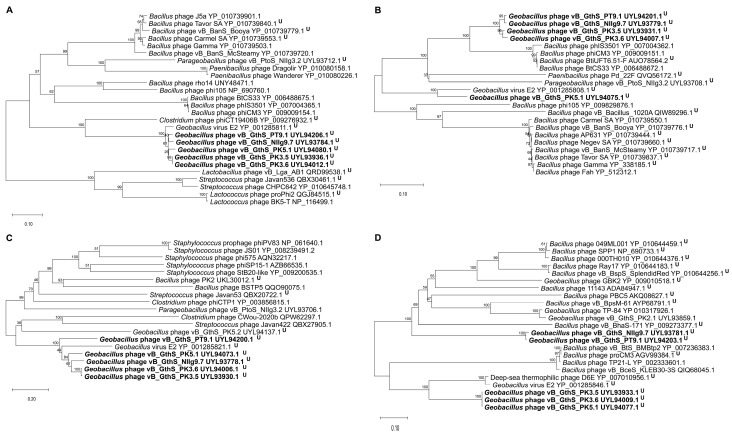
Neighbor-joining tree analysis based on the alignment of the amino acid sequences of isolated thermophilic bacteriophages: (**A**) major capsid protein, (**B**) terminase large subunit, (**C**) tape measure protein (TMP), and (**D**) helicase. The percentage of replicate trees in which the associated taxa clustered together in the bootstrap test is shown next to the branches. U—unclassified bacteriophages within the class *Caudoviricetes*.

In order to determine the phylogenetic relationship among isolated thermophilic bacteriophages and their closest relatives to date, a comparison of the individual genes most often used for the analysis of the evolutionary relationships among bacteriophages [47] was carried out. The phylogenetic trees based on the alignment of the PT9.1, NIIg9.7, PK3.5, PK3.6, and PK5.1 major capsid protein, terminase large subunit, tape measure protein, replicative helicase, and amino acid sequences with those returned by BLASTP homology searches were constructed (Figure 5). It was demonstrated that, in most cases, isolated *Geobacillus* bacteriophages were phylogenetically the most closely interconnected to each other but distant from other phages and occupied a somewhat intermediate position among unclassified siphoviruses within the class *Caudoviricetes*.

**Figure 6:**
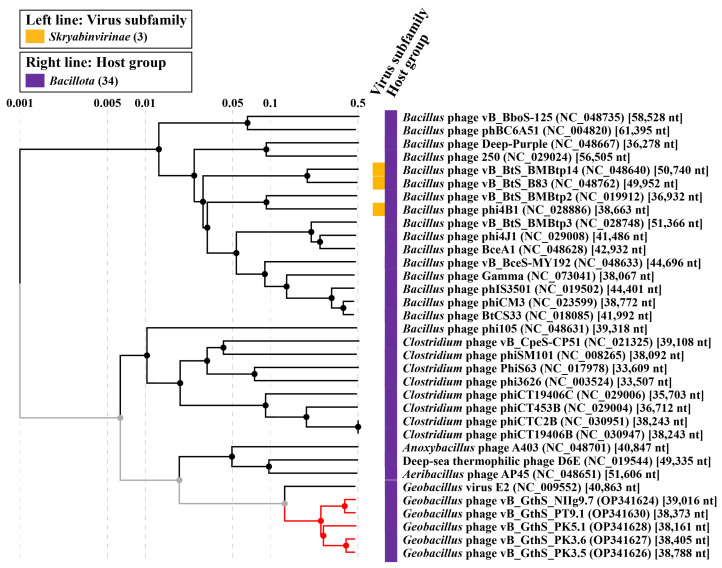
ViPTree-generated proteomic tree of isolated thermophilic bacteriophages and dsDNA viruses represented in the rectangular view. The tree is constructed by BIONJ based on genomic distance matrixes, and mid-point rooted. Branch lengths are logarithmically scaled from the root of the entire proteomic tree. The numbers at the top represent the log-scaled branch lengths based on the SG (normalized tBLASTx scores) values.

To obtain a more detailed picture of the phylogenetic relationships of PT9.1, NIIg9.7, PK3.5, PK3.6, PK5.1, and their closest relatives, a comparative total proteome comparison was performed using the ViPTree web service. Based on the whole-proteome alignment of isolated *Geobacillus* phages and their closest relatives, it was demonstrated that phage PT9.1 is the most closely related to NIIg9.7, whereas the closest relative of PK3.5 was PK3.6. Phage PK5.1 was in between PT9.1-NIIg9.7 and PK3.5-PK3.6 (Figure 6). Bacteriophages of this study were the most closely related to unclassified *Geobacillus* virus GVE2 (NC_009552). However, the phylogenetic relationship between phages characterized in this study and virus GVE2 was distant, suggesting that PT9.1, NIIg9.7, PK3.5, PK3.6, and PK5.1 formed a not yet identified cluster of phages within the siphoviruses.

**Figure 7:**
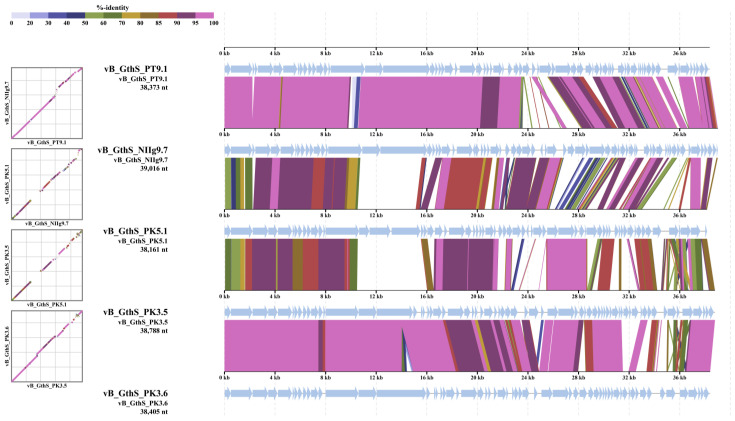
ViPTree-generated whole-proteome alignment of isolated thermophilic bacteriophages. Colored lines in the alignment indicate tBLASTx results (E-value < 0.01). Positions of each sequence are automatically adjusted (i.e., circularly permuted and reverse-stranded) for clear representation of collinearity among genomes.

To determine the most homologous regions in the genomes of isolated thermophilic phages, genome alignment was performed by using ViPTree. Genomes of all bacteriophages shared several regions of nucleotide similarity that covered the essential structural and virion morphogenesis protein-encoding genes, as well as genes related to lysis and DNA metabolism and modification (Figure 7).

**Figure 8:**
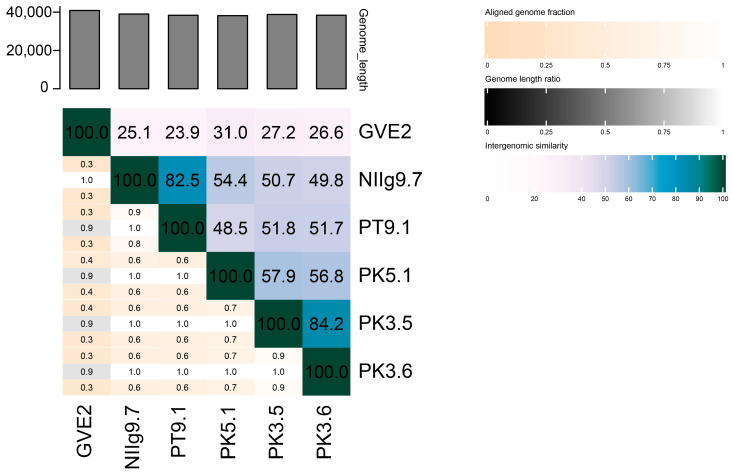
The whole-genome comparison and clustering of phages PT9.1, NIIg9.7, PK5.1, PK3.5, PK3.6, and their closest relative—*Geobacillus* virus GVE2. The comparison and clustering were performed with the use of VIRIDIC. Different shades of blue in the right half of the heatmap represent different intergenomic similarities (%) between the genomes of each pair compared, as indicated above the heatmap and specified by numbers. The left half of the heatmap shows three indicator values for each genome pair: aligned fraction of genome one for the genome in this row (top value), genome length ratio for the two genomes in this pair (middle value), and aligned fraction of genome two for the genome in this column (bottom value).

Nevertheless, the nucleotide-based virus overall nucleotide sequence identity in-between isolated *Geobacillus* phages and their closest relatives was calculated using VIRIDIC (Figure 8). It was demonstrated that the highest identity (84.2%) was between phages PK3.5 and PK3.6. Similarly, the identity of PT9.1 vs. NIIg9.7 was 82.5%, whereas phage PK5.1 demonstrated 57.9%, 56.8%, 54.4%, and 48.5% identity with PK3.5, PK3.6, NIIg9.7, and PT9.1, accordingly. In addition, of all the phages studied in this work, PK5.1 shared the highest overall nucleotide identity with other viruses sequenced to date, which is 31.0% identity with *Geobacillus* virus GVE2. Thus, it seemed that the bacteriophages of this study were phylogenetically the most closely related to each other and potentially represent a new cluster of thermophilic siphoviruses.

According to the Bacterial and Archaeal Viruses Subcommittee (BAVS) of the International Committee on Taxonomy of Viruses (ICTV), two phages are assigned to the same species if their genomes are more than 95% identical, while a genus is described as a cohesive group of viruses sharing a high degree (>70%) of nucleotide identity of the full genome length [48]. Following this and based on the results of the comparative genome sequence analysis performed during this study, we considered that all bacteriophages of this study represent new species. Moreover, PK3.5 together with PK3.6, as well as PT9.1 together with NIIg9.7, and a singleton PK5.1 could not be classified to any genus currently recognized by ICTV, and likely represent three new genera within the siphoviruses.

## 4. Discussion

*Geobacillus* spp. are widely distributed throughout environments, and the high potential of these bacteria and/or their enzymes in various biotechnological, industrial, or medical applications had been reported [4,5,6]. On the other hand, *G. stearothermophilus* can cause flat sour spoilage in low-acid canned foods [49]. In addition, *G. stearothermophilus*, *G. thermoleovorans*, and *G. thermodenitrificans* often form biofilms on different abiotic surfaces, which cause significant financial losses in the food industry [50,51,52]. Thus, a number of publications on lytic enzymes from *Geobacillus* phages, which demonstrated antimicrobial activity against their hosts and other detrimental bacterial strains, have been reported. The vast majority of these reports characterized viral endolysins [15,53,54,55,56,57].

Genomic analysis of *Geobacillus* bacteriophages PT9.1, NIIg9.7, PK5.1, PK3.5, and PK3.6 revealed that the lysis cassette of all these phages consisted of three lytic enzymes: two potential holins and one endolysin. Endolysins of bacteriophages of this study were N-acetylmuramoyl-L-alanine amidases containing N-terminal MurNAc-LAA and C-terminal SPOR superfamily conserved domains, and one of their closest BLASTp homologs was a well-characterized endolysin (YP_001285830.1) from *Geobacillus* virus GVE2. It was demonstrated that the GVE2 recombinant endolysin was thermostable, exhibited good tolerances at acid pH values, and played important roles in the lysis process of the host cells [53,54]. Bioinformatics analysis revealed that the identity in the amino acid level of endolysins from phages of this study to GVE2 endolysin ranged from 59.15 to 60.41% (phages PK3.6 and PK5.1, accordingly). Thus, the endolysins from phages of this study could also be attractive tools in applications in molecular biology as well as industries, although more detailed studies would be needed to confirm this.

In contrast to endolysins, to our knowledge, only one depolymerase from *Geobacillus* phages, which is a thermostable capsule depolymerase from phage TP-84 [58], has been characterized in detail to date. Phage-encoded depolymerases are collectively referred to as enzymes able to degrade polymeric substances found in the bacterial cell surface, such as polysaccharides, or present in bacterial biofilms [59]. The depolymerase activity is commonly identified by a constantly increasing halo zone surrounding the phage plaques [60,61]. The vast majority of phage depolymerases are encoded in the same open reading frame of phage structural proteins (mostly on tail spikes, tail fibers, and base plates) or in close proximity to those genes, and thus are considered structural proteins [59,62].

The morphology of plaques (clear central part surrounded by a turbid halo zone) formed by *Geobacillus* phages PT9.1, NIIg9.7, PK5.1, PK3.5, and PK3.6 suggested that all viruses of this study possessed depolymerase activity. Bioinformatics analysis revealed that potential candidates of this activity were tail fiber proteins encoded by PT9.1 ORF18, NIIg9.7 ORF17, PK5.1 ORF17, PK3.5 ORF16, and PK3.6 ORF16. BLASTp analysis demonstrated that all aforementioned proteins contain N-terminal Prophage_tail (pfam06605) superfamily domain presented in phage tail proteins that are probably acting as endopeptidases, whereas diverse conserved domains were encoded in the C-terminus of the aforementioned proteins (Figure S3). Potential depolymerases of phages PT9.1, NIIg9.7, and PK5.1 possessed C-terminal Peptidase_S74 (pfam13884) domain, which is specific for depolymerases acting as sialidases [59]. In contrast, gp16 of phage PK3.5 and gp16 of phage PK3.6 encoded central tolA_full (TIGR02794) and C-terminal choice_anch_G (NF033766) and (PHA02515), respectively, conserved domains, which had never been characterized as domains specific to depolymerases. However, HHpred analysis demonstrated that gp16 of PK3.5 and gp16 of PK3.6 had the best hits to tail-associated lysin (6V8I_CE) of *Staphylococcus* phage 80alpha (probability 99.98% and E-value 7.6 × 10^−21^; probability 99.88% and E-value 1.5 × 10^−20^, respectively). Thus, it is likely that all five aforementioned tail fiber proteins of the isolated *Geobacillus* phages could have potential depolymerase activity, although experimental validation would be needed to confirm this hypothesis.

The results of the host range determination experiments confirmed that isolated thermophilic bacteriophages, especially PK3.6 and PK5.1, were very specific for their hosts (considering that 41 out of the 46 tested bacterial strains were isolated from the same environment and at the same time as the phages, and that all tested strains belonged to the *Bacillus*-group bacteria, including 20 strains of *G. thermodenitrificans*). While many well-studied model phages seemed to exhibit a narrow host range, recent ecological and metagenomics studies indicated that phages may have specificities that ranged from narrow to broad [63]. However, it is still an open question what factors determine this specificity, especially in the case of thermophilic bacteriophages. It is known that viral host range could be determined by a number of molecular mechanisms [63,64], and it is likely that the initial stage of phage infection could be one of the most important.

The PT9.1 gp18, NIIg9.7 gp17, PK5.1 gp17, PK3.5 gp16, and PK3.6 gp16 were tail fiber proteins, which, in our prediction, could play a significant role in the initial steps of host recognition and adsorption. Summarizing the results of the bioinformatics analysis, PT9.1 gp18 shared a high identity with NIIg9.7 gp17, as well as PK3.5 gp16 with PK3.6 gp16; meanwhile, PK5.1 gp17 had no close homology at the amino acid level to the aforementioned proteins. In comparison, the host range determination experiments revealed that phages PT9.1 and NIIg9.7 were active against nine *G. thermodenitrificans* strains (including seven strains that are the same for both phages), whereas PK5.1 and PK3.6 infected only two *G. thermodenitrificans* strains (including one strain that is the same for both phages). On the other hand, despite the high similarity of PK3.5 gp16 and PK3.6 gp16, phage PK3.5 infected four *G. thermodenitrificans* strains. Thus, it was likely that different profiles of phage hosts may be determined by small differences in the amino acid sequences of tail fiber proteins and/or by other viral proteins potentially included in phage–host interaction or even later stages of the phage replication cycle. Further studies, however, would be needed to gain a more comprehensive understanding of the interactions between thermophilic *Geobacillus* phages and their hosts.

## 5. Conclusions

In conclusion, we showed that *Geobacillus*-infecting bacteriophages PT9.1, NIIg9.7, PK5.1, PK3.5, and PK3.6 are thermophilic siphoviruses possessing potential depolymerase activity and diverse host profiles. In addition, our results indicate that the isolated phages are substantially distinct from all of the previously described phages and may be considered representatives of three novel genera within the siphoviruses.

## Figures and Tables

**Table 1:** Morphological features and plaque morphology of isolated thermophilic bacteriophages.

Phage	Phage Morphology			Plaque Morphology *	
	Head Diameter (nm)	Tail Length (nm)	Tail Width (nm)	Diameter of a Clear Center (mm)	Diameter of Plaque with a Halo Zone (mm)
PT9.1	62.72 ± 2.34	143.15 ± 8.47	10.68 ± 1.62	3.21 ± 0.22	9.43 ± 0.73
NIIg9.7	63.81 ± 3.95	143.16 ± 4.19	9.93 ± 1.62	3.04 ± 0.31	11.42 ± 0.75
PK5.1	62.91 ± 3.20	145.74 ± 14.88	10.10 ± 1.42	3.67 ± 0.50	7.20 ± 1.23
PK3.5	65.18 ± 3.27	149.98 ± 15.01	10.84 ± 1.92	1.45 ± 0.33	4.92 ± 0.98
PK3.6	66.93 ± 4.24	137.32 ± 5.75	9.41 ± 1.84	3.01 ± 0.51	8.42 ± 0.62

*—the morphological characteristics of the plaque-forming units were monitored after 24 h of incubation on LB agar plates at 55 °C, using *Geobacillus thermodenitrificans* strains PT-9 (phage PT9.1), NIIg-9 (phage NIIg9.7), PK-5 (phage PK5.1), and PK-3 (phages PK3.5 and PK3.6) as the host bacteria.

**Table 2:** Genomic characteristics of isolated thermophilic bacteriophages.

Phage	Genome Size (bp)	GC Content (%)	Coding Capacity (%)	N° of ORFs	Best Sequence Alignment *	Identity (%) (Query Coverage (%))
PT9.1	38,373	43.9	94.2	75	Geobacillus phage vB_GthS_NIIg9.7	97.04 (85)
NIIg9.7	39,016	44.4	93.9	76	Geobacillus phage vB_GthS_PT9.1	97.04 (83)
PK5.1	38,161	43.6	92.4	64	Geobacillus phage vB_GthS_PK3.5	89.31 (59)
PK3.5	38,788	43.5	92.7	76	Geobacillus phage vB_GthS_PK3.6	97.60 (84)
PK3.6	38,405	44.8	92.0	68	Geobacillus phage vB_GthS_PK3.5	97.60 (85)

*—data obtained from BLASTn performed in the NCBI database.

## Data Availability

The complete genome sequences of *Geobacillus* bacteriophages of this study are available in the EMBL nucleotide sequence database under accession numbers OP341630 (phage vB_GthS_PT9.1), OP341624 (phage vB_GthS_NIIg9.7), OP341628 (phage vB_GthS_PK5.1), OP341626 (phage vB_GthS_PK3.5), and OP341627 (phage vB_GthS_PK3.6). The sequences of the PCR-amplified 16S rRNA gene fragments of *Bacillus*-group bacteria isolated during this study are available in the EMBL nucleotide sequence database under accession numbers presented in Table S1.

## Supplementary Materials

The following supporting information can be downloaded at: https://www.mdpi.com/article/10.3390/v15081691/s1, Figure S1: the morphology of colonies of isolated bacterial strains after 24 h of incubation on LB agar medium at 55 °C; Figure S2: bacterial growth temperature ranges; Figure S3: comparison of conserved domains in potential depolymerase genes of thermophilic bacteriophages PT9.1, NIIg9.7, PK5.1, PK3.5, and PK3.6; Table S1: bacterial strains used in this study to determine the host range of the bacteriophages; Table S2: the host range of the *Geobacillus* bacteriophages; Table S3: summarized information of assembled viral contigs and nucleotide sequences of primers used to confirm the completeness of the assembled contigs.; Table S4: PT-9.1 ORFs with homologs in other viruses or cellular organisms; Table S5: NIIg9.7 ORFs with homologs in other viruses or cellular organisms; Table S6: PK5.1 ORFs with homologs in other viruses or cellular organisms; Table S7: PK3.5 ORFs with homologs in other viruses or cellular organisms; Table S8: PK3.6 ORFs with homologs in other viruses or cellular organisms; Table S9: viral structural proteins identified by Mass Spectrometry.
