# Supplementary material for: Geobacillus Bacteriophages from Compost Heaps: Representatives of Three New Genera within Thermophilic Siphoviruses
Source: Viruses. 2023 Aug 4;15(8):1691. doi: 10.3390/v15081691 (PMC10459684; doi:10.3390/v15081691)
Supplement: Supplementary file 1 [file viruses-15-01691-s001.zip › viruses-2536460-supplementary.pdf]

Supplementary material

Geobacillus bacteriophages from compost heaps: representatives of three new genera within thermophilic siphoviruses

Eugenijus Šimoliūnas [1,2*], Monika Šimoliūnienė [1], Gintarė Laskevičiūtė [1], Kotryna Kvederavičiūtė [3], Martynas Skapas [4], Algirdas Kaupinis [5], Mindaugas Valius [5], Rolandas Meškys [1] and Nomeda Kuisienė [2]

1 Department of Molecular Microbiology and Biotechnology, Institute of Biochemistry, Life Sciences Center, Vilnius University, Saulėtekio av. 7, Vilnius LT-10257, Lithuania; eugenijus.simoliunas@bchi.vu.lt (E.Š.); monika.simoliuniene@gmc.vu.lt (M.Š.); gintare.laskeviciute@gmail.com (G.L.); rolandas.meskys@bchi.vu.lt (R.M.)
2 Department of Microbiology and Biotechnology, Institute of Bioscience, Life Sciences Center, Vilnius Uni-versity, Saulėtekio av. 7, Vilnius LT-10257, Lithuania; eugenijus.simoliunas@bchi.vu.lt (E.Š.); nomeda.kuisiene@gf.vu.lt (N.K.)
3 Department of Biological DNA Modification, Institute of Biotechnology, Life Sciences Center, Vilnius Uni-versity, Saulėtekio av. 7, Vilnius LT-10257, Lithuania; kotryna.kvederaviciute@mif.vu.lt (K.K.)
4 Department of Characterisation of Materials Structure, Center for Physical Sciences and Technology, Sau-letekio av. 3, LT-10257 Vilnius, Lithuania; martynas.skapas@ftmc.lt (M.S.)
5 Proteomics Centre, Institute of Biochemistry, Life Sciences Center, Vilnius University, Saulėtekio av. 7, Vil-nius LT-10257, Lithuania; algirdas.kaupinis@gf.vu.lt (A.K.); mindaugas.valius@bchi.vu.lt (M.V.)
* Correspondence: eugenijus.simoliunas@bchi.vu.lt (E.Š.); Tel.: +3706-507-0467

Table S1. Bacterial strains used in this study to determine the host range of the bacteriophages.

Strain<br>(the accession number of PCR-amplified 16S rRNA gene sequence)	Relevant characteristics	Source
Aeribacillus pallidus DSM 3670^T	type strain	DSMZ
Aeribacillus pallidus strain SIII-6 (OQ561262)	environmental isolate	this study
Aeribacillus pallidus strain PT-10 (OQ561260)	environmental isolate	this study
Aeribacillus pallidus strain NIIg-6 (OQ561255)	environmental isolate	this study
Aeribacillus pallidus strain NIIg-7 (OQ561256)	environmental isolate	this study
Aeribacillus pallidus strain NIIg-5 (OQ561254)	environmental isolate	this study
Aeribacillus pallidus strain PK-8 (OQ561259)	environmental isolate	this study
Aeribacillus pallidus strain P-5 (OQ561258)	environmental isolate	this study
Aeribacillus pallidus strain SIII-3 (OQ561261)	environmental isolate	this study
Aeribacillus pallidus strain NIIg-8 (OQ561257)	environmental isolate	this study
Brevibacillus borstelensis strain P8 (OQ561264)	environmental isolate	this study
Brevibacillus borstelensis strain P-4 (OQ561263)	environmental isolate	this study
Geobacillus lituanicus DSM 15325^T	type strain	DSMZ
Geobacillus stearothermophilus DSM 22^T	type strain	DSMZ
Geobacillus thermodenitrificans DSM 465^T	type strain	DSMZ
Geobacillus thermodenitrificans strain PK-6 (OQ561277)	environmental isolate	this study
Geobacillus thermodenitrificans strain SIII-1 (OQ561283)	environmental isolate	this study
Geobacillus thermodenitrificans strain NIIg-1 (OQ561265)	environmental isolate	this study
Geobacillus thermodenitrificans strain PK-1-10 (OQ561273)	environmental isolate	this study
Geobacillus thermodenitrificans strain PT-4 (OQ561280)	environmental isolate	this study
Geobacillus thermodenitrificans strain NIIg-2 (OQ561266)	environmental isolate	this study
Geobacillus thermodenitrificans strain NIIg-9 (OQ561267)	environmental isolate	this study
Geobacillus thermodenitrificans strain PK-11 (OQ561278)	environmental isolate	this study
Geobacillus thermodenitrificans strain P-1 (OQ561270)	environmental isolate	this study
Geobacillus thermodenitrificans strain PK-2 (OQ561274)	environmental isolate	this study
Geobacillus thermodenitrificans strain PT-9 (OQ561282)	environmental isolate	this study
Geobacillus thermodenitrificans strain PT-5 (OQ561281)	environmental isolate	this study
Geobacillus thermodenitrificans strain PT-3 (OQ561279)	environmental isolate	this study
Geobacillus thermodenitrificans strain NIIg-11 (OQ561269)	environmental isolate	this study
Geobacillus thermodenitrificans strain P-2 (OQ561271)	environmental isolate	this study
Geobacillus thermodenitrificans strain PK-5 (OQ561276)	environmental isolate	this study
Geobacillus thermodenitrificans strain NIIg-10 (OQ561268)	environmental isolate	this study
Geobacillus thermodenitrificans strain PK-1 (OQ561272)	environmental isolate	this study
Geobacillus thermodenitrificans strain PK-3 (OQ561275)	environmental isolate	this study
Parageobacillus caldoxylosilyticus strain P-4-70 (OQ561284)	environmental isolate	this study
Parageobacillus caldoxylosilyticus strain PK-7 (OQ561287)	environmental isolate	this study
Parageobacillus caldoxylosilyticus strain PK-4 (OQ561286)	environmental isolate	this study
Parageobacillus caldoxylosilyticus strain SIII-4 (OQ561288)	environmental isolate	this study
Parageobacillus caldoxylosilyticus strain P-6 (OQ561285)	environmental isolate	this study
Parageobacillus thermoglucosidasius strain P-3 (OQ561289)	environmental isolate	this study
Parageobacillus thermoglucosidasius strain SIII-8 (OQ561290)	environmental isolate	this study
Parageobacillus toebii strain NIIg-3 (OQ561291)	environmental isolate	this study
Peribacillus butanolivorans DSM 18929^T	type strain	DSMZ
Ureibacillus terrenus strain NIIg-4 (OQ561292)	environmental isolate	this study
Ureibacillus thermosphaericus strain PK-2-65 (OQ561294)	environmental isolate	this study
Ureibacillus thermosphaericus strain P1 (OQ561293)	environmental isolate	this study

Table S2. Summarized information of assembled viral contigs and nucleotide sequences of primers used to confirm the completeness of the assembled contigs. Contigs were assembled using SPAdes packages version 3.13.1. BBMAP package (v38.96) was used to evaluate mapping rate and coverage.

Phage	Size of assembled contig (bp)	Overall mapping rate	Average coverage	Primers used to confirm the completeness of the assembled contigs	
				Name	Sequence (5′--3′)
PT9.1	38,450	98.389	546.829	PT9.1F<br>PT9.1R	GTCGGTTCAGATGGCAACCCGTAG<br>CCTACTGCCCTGTATGCGGCTATG
NIIg9.7	39,093	98.410	537.144	NIIg9.7F<br>NIIg9.7R	GGACGATGTGGCTGAAGGTGTTC<br>CGTCGGCGTAGCCTCATCAGAC
PK5.1	38,238	98.010	539.642	PK5.1F<br>PK5.1R	GGTGAGATTCACCATGTCCGGCAC<br>GATACGTCAACGATACGTTCCACG
PK3.5	38,865	98.724	545.990	PK3.5F<br>PK3.5R	GCGGAAGGAGCGACATCGACATAC<br>GGTTTCGAGATCGTTCATCACGG
PK3.6	38,482	98.696	539.575	PK3.6F<br>PK3.6R	GGATTCGAGGTAGAAGGCGAGC<br>GGAACCAATTGACGTATCGCACG

Table S3. The host range of the *Geobacillus* bacteriophages.

Species	Phage / Strain	PT9.1	NIIg9.7	PK5.1	PK3.5	PK3.6
Aeribacillus pallidus	DSM 3670^T					
	SIII-6					
	PT-10					
	NIIg-6					
	NIIg-7					
	NIIg-5					
	PK-8					
	P-5					
	SIII-3					
	NIIg-8					
Brevibacillus borstelensis	P8					
	P-4					
Geobacillus lituanicus	DSM 15325^T					
Geobacillus stearothermophilus	DSM 22^T					
Geobacillus thermodenitrificans	DSM 465^T					
	PK-6				[blue]	
	SIII-1	[blue]	[blue]			
	NIIg_1	[blue]	[blue]			
	PK-1-10	[blue]	[blue]			
	PT-4					
	NIIg-2					
	NIIg-9	[blue]	[blue] *			
	PK-11	[yellow]				
	P-1	[blue]	[blue]			
	PK-2					
	PT-9	[blue] *	[blue]			
	PT-5					
	PT-3	[yellow]	[yellow]			
	NIIg-11				[blue]	
	P-2				[yellow]	
	PK-5	[blue]		[blue] *		
	NIIg-10		[yellow]			[blue]
	PK-1					
	PK-3		[blue]	[blue]	[blue] *	[blue] *
Parageobacillus caldoxylosilyticus	P-4-70					
	PK-7					
	PK-4					
	SIII-4					
	P-6					
Parageobacillus thermoglucosidasius	P-3					
	SIII-8					
Parageobacillus toebii	NIIg-3					
Peribacillus butanolivorans	DSM 18929^T					
Ureibacillus terrenus	NIIg-4					
Ureibacillus thermosphaericus	PK-2-65					
	P1					

Colors: blue, complete lysis; yellow, partial lysis. * Host strain used for the bacteriophage isolation.

Table S4. PT-9.1 ORFs with homologues in other viruses or cellular organisms.

PT-9.1 ORF (position in genome)	Predicted function (protein length (aa))	Significant match (protein length (aa))	Identity/ similarity (%) (length of the overlapping segment (aa))	E value
ORF01<br>(16 -> 510)	terminase small subunit (164)	UYL93951.1 terminase small subunit<br>*Geobacillus* phage vB_GthS_PK3.5 (164)	99/100 (164)	9e-114
ORF02<br>(507 -> 2,210)	terminase large subunit (567)	UYL93779.1 terminase large subunit<br>*Geobacillus* phage vB_GthS_NIIg9.7 (567)	99/99 (567)	0.0
ORF03<br>(2,233 -> 2,397)	hypothetical protein (54)	WP_157778131.1 hypothetical protein<br>*Parageobacillus thermoglucosidasius* (54)	100/100 (54)	3e-30
ORF04<br>(2,413 -> 2,607)	hypothetical protein (64)	UYL93835.1 hypothetical protein<br>*Geobacillus* phage vB_GthS_NIIg9.7 (64)	92/95 (64)	1e-33
ORF05<br>(2,641 -> 3,873)	portal protein (410)	UYL93783.1 portal protein<br>*Geobacillus* phage vB_GthS_NIIg9.7 (410)	99/100 (410)	0.0
ORF06<br>(3,873 -> 4,607)	Clp protease (244)	UYL93941.1 Clp protease<br>*Geobacillus* phage vB_GthS_PK3.5 (245)	99/100 (244)	4e-177
ORF07<br>(4,649 -> 5,764)	major capsid protein (371)	UYL93936.1 major capsid protein<br>*Geobacillus* phage vB_GthS_PK3.5 (371)	98/98 (371)	0.0
ORF08<br>(5,810 -> 5,956)	hypothetical protein (48)	YP_001285812.1 hypothetical protein<br>*Geobacillus* virus E2 (48)	98/100 (48)	5e-26
ORF09<br>(5,953 -> 6,240)	head-tail connector protein (95)	UYL94115.1 head-tail connector protein<br>*Geobacillus* phage vB_GthS_PK5.1 (95)	99/98 (95)	1e-61
ORF10<br>(6,237 -> 6,578)	head closure protein (113)	UYL93809.1 head closure protein<br>*Geobacillus* phage vB_GthS_NIIg9.7 (113)	97/99 (113)	9e-77
ORF11<br>(6,559 -> 6,939)	putative tail-component (126)	UYL94104.1 putative tail-component<br>*Geobacillus* phage vB_GthS_PK5.1 (126)	98/99 (126)	1e-85
ORF12<br>(6,941 -> 7,261)	putative tail-component (106)	UYL93813.1 putative tail-component<br>*Geobacillus* phage vB_GthS_NIIg9.7 (106)	98/99 (106)	2e-69
ORF13<br>(7,267 -> 7,845)	major tail protein (192)	UYL93793.1 major tail protein<br>*Geobacillus* phage vB_GthS_NIIg9.7 (192)	99/100 (192)	1e-139
ORF14<br>(7,849 -> 8,187)	hypothetical protein (112)	UYL93811.1 hypothetical protein<br>*Geobacillus* phage vB_GthS_NIIg9.7 (112)	98/100 (112)	9e-75
ORF15<br>(8,138 -> 8,395)	hypothetical protein (85)	UYL93822.1 hypothetical protein<br>*Geobacillus* phage vB_GthS_NIIg9.7 (85)	99/100 (85)	7e-54
ORF16<br>(8,409 -> 11,105)	tape measure protein (898)	UYL93778.1 tape measure protein<br>*Geobacillus* phage vB_GthS_NIIg9.7 (892)	75/81 (902)	0.0
ORF17<br>(11,118 -> 12,551)	distal tail protein (477)	UYL93780.1 distal tail protein<br>*Geobacillus* phage vB_GthS_NIIg9.7 (477)	98/99 (477)	0.0
ORF18<br>(12,548 -> 16,261)	tail fiber protein (1237)	UYL93777.1 tail fiber protein<br>*Geobacillus* phage vB_GthS_NIIg9.7 (1237)	98/98 (1237)	0.0
ORF19<br>(16,258 -> 16,674)	hypothetical protein (138)	UYL93803.1 hypothetical protein<br>*Geobacillus* phage vB_GthS_NIIg9.7 (138)	100/100 (137)	2e-91
ORF20<br>(16,674 -> 16,901)	hypothetical protein (75)	UYL93827.1 hypothetical protein<br>*Geobacillus* phage vB_GthS_NIIg9.7 (75)	99/100 (75)	3e-45
ORF21<br>(16,939 -> 17,157)	hemolysin (72)	UYL93829.1 hemolysin<br>*Geobacillus* phage vB_GthS_NIIg9.7 (72)	99/100 (72)	3e-41
ORF22<br>(17,157 -> 17,417)	holin (86)	UYL93821.1 holin<br>*Geobacillus* phage vB_GthS_NIIg9.7 (86)	99/100 (86)	3e-50
ORF23<br>(17,417 -> 18,103)	N-acetylmuramoyl-L-alanine amidase (228)	YP_001425618.1 N-acetylmuramoyl-L-alanine amidase<br>Svunavirus sv1 (226)	86/90 (202)	3e-105

ORF24 (18,181 -> 18,444)	hypothetical protein (87)	UYL93966.1 hypothetical protein *Geobacillus* phage vB_GthS_PK3.5 (95)	90/95 (87)	1e-50
ORF25 (18,598 -> 19,860)	FtsK/SpoIIIE family protein (420)	UYL93782.1 FtsK/SpoIIIE family protein *Geobacillus* phage vB_GthS_NIIg9.7 (420)	99/98 (420)	0.0
ORF26 (19,766 -> 20,383)	replication/relaxation protein (205)	UYL93945.1 replication/relaxation protein *Geobacillus* phage vB_GthS_PK3.5 (205)	98/99 (205)	3e-147
ORF27 (20,506 -> 20,694)	XRE family transcriptional regulator (62)	UYL93837.1 XRE family transcriptional regulator *Geobacillus* phage vB_GthS_PK3.5 (62)	100/100 (62)	1e-35
ORF28 (20,737 -> 20,943)	hypothetical protein (68)	UYL93833.1 hypothetical protein *Geobacillus* phage vB_GthS_NIIg9.7 (68)	99/98 (68)	5e-41
ORF29 (20,957 -> 21,190)	hypothetical protein (77)	UYL94048.1 hypothetical protein *Geobacillus* phage vB_GthS_PK3.6 (84)	97/97 (76)	2e-45
ORF30 (21,177 -> 21,386)	hypothetical protein (69)	UYL93831.1 hypothetical protein *Geobacillus* phage vB_GthS_NIIg9.7 (69)	99/98 (69)	1e-37
ORF31 (21,391 -> 21,981)	ERF family protein (196)	YP_007236371.1 ERF family protein *Bacillus* phage vB_BtS_BMBtp2 (218)	54/72 (138)	6e-48
ORF32 (21,978 -> 22,106)	hypothetical protein (42)	UYL94000.1 hypothetical protein *Geobacillus* phage vB_GthS_PK3.5 (42)	100/100 (42)	4e-19
ORF33 (22,094 -> 22,309)	hypothetical protein (71)	UYL93830.1 hypothetical protein *Geobacillus* phage vB_GthS_NIIg9.7 (71)	99/98 (71)	6e-43
ORF34 (22,306 -> 22,419)	hypothetical protein (37)	UYL93850.1 hypothetical protein *Geobacillus* phage vB_GthS_NIIg9.7 (37)	97/97 (37)	9e-16
ORF35 (22,416 -> 22,757)	hypothetical protein (113)	UYL93810.1 hypothetical protein *Geobacillus* phage vB_GthS_NIIg9.7 (113)	100/100 (113)	2e-73
ORF36 (22,750 -> 22,908)	hypothetical protein (52)	UYL93843.1 hypothetical protein *Geobacillus* phage vB_GthS_NIIg9.7 (52)	100/100 (52)	2e-30
ORF37 (22,926 -> 23,306)	hypothetical protein (126)	UYL93807.1 hypothetical protein *Geobacillus* phage vB_GthS_NIIg9.7 (126)	99/99 (126)	1e-84
ORF38 (23,303 -> 23,611)	hypothetical protein (102)	UYL94040.1 hypothetical protein *Geobacillus* phage vB_GthS_PK3.6 (102)	97/100 (102)	1e-66
ORF39 (23,608 -> 24,150)	hypothetical protein (180)	UYL94023.1 hypothetical protein *Geobacillus* phage vB_GthS_PK3.6 (180)	96/98 (180)	2e-115
ORF40 (24,373 -> 24,603)	XRE family transcriptional regulator (76)	UYL93977.1 XRE family transcriptional regulator *Geobacillus* phage vB_GthS_PK3.5 (76)	100/100 (76)	2e-46
ORF41 (24,913 -> 25,884)	DNA replication initiation protein (323)	UYL93785.1 DNA replication initiation protein *Geobacillus* phage vB_GthS_NIIg9.7 (280)	49/64 (284)	1e-66
ORF42 (25,877 -> 26,164)	loader and inhibitor of replicative helicase (95)	UYL93818.1 loader and inhibitor of replicative helicase *Geobacillus* phage vB_GthS_NIIg9.7 (95)	100/100 (95)	4e-64
ORF43 (26,148 -> 27,419)	replicative DNA helicase (423)	UYL93781.1 replicative DNA helicase *Geobacillus* phage vB_GthS_NIIg9.7 (423)	99/99 (423)	0.0
ORF44 (27,416 -> 27,874)	hypothetical protein (152)	UYL93798.1 hypothetical protein *Geobacillus* phage vB_GthS_NIIg9.7 (152)	97/98 (152)	2e-102
ORF45 (27,889 -> 28,395)	HNH endonuclease (168)	UYL93795.1 HNH endonuclease *Geobacillus* phage vB_GthS_NIIg9.7 (178)	86/91 (168)	5e-90
ORF46 (28,373 -> 29,095)	HNH endonuclease (240)	YP_009151515.1 HNH homing endonuclease *Bacillus* phage Pascal (250)	40/56 (183)	4e-34
ORF47 (29,092 -> 29,211)	hypothetical protein (39)	UYL93849.1 hypothetical protein *Geobacillus* phage vB_GthS_NIIg9.7 (40)	95/100 (38)	2e-15
ORF48 (29,261 -> 29,680)	single-stranded DNA binding protein (139)	UYL93802.1 single-stranded DNA binding protein *Geobacillus* phage vB_GthS_NIIg9.7 (139)	91/94 (139)	6e-88

ORF49 (29,695 -> 29,892)	hypothetical protein (65)	YP_009010523.1 hypothetical protein *Geobacillus* phage GBK2 (65)	94/100 (65)	7e-28
ORF50 (29,910 -> 30,350)	DNA N-6-adenine-methyltransferase (146)	UYL93800.1 DNA N-6-adenine-methyltransferase *Geobacillus* phage vB_GthS_NIIg9.7 (146)	99/100 (146)	9e-106
ORF51 (30,347 -> 30,460)	hypothetical protein (37)	UYL93851.1 hypothetical protein *Geobacillus* phage vB_GthS_NIIg9.7 (37)	97/100 (37)	3e-15
ORF52 (30,457 -> 30,639)	hypothetical protein (60)	UYL93836.1 hypothetical protein *Geobacillus* phage vB_GthS_NIIg9.7 (63)	100/100 (60)	5e-36
ORF53 (30,641 -> 30,826)	hypothetical protein (61)	UYL93987.1 hypothetical protein *Geobacillus* phage vB_GthS_PK3.5 (61)	98/98 (61)	2e-30
ORF54 (30,840 -> 31,145)	hypothetical protein (101)	UYL93815.1 hypothetical protein *Geobacillus* phage vB_GthS_NIIg9.7 (101)	95/97 (101)	2e-65
ORF55 (31,123 -> 31,653)	dUTP diphosphatase (176)	YP_008129907.1 dUTPase *Paenibacillus* phage PG1 (180)	42/56 (185)	3e-39
ORF56 (31,658 -> 31,822)	hypothetical protein (54)	YP_007010964.1 hypothetical protein Deep-sea thermophilic phage D6E (59)	96/100 (53)	2e-25
ORF57 (31,832 -> 32,143)	holliday junction resolvase (103)	UYL93814.1 holliday junction resolvase *Geobacillus* phage vB_GthS_NIIg9.7 (103)	98/100 (103)	1e-64
ORF58 (32,127 -> 32,252)	hypothetical protein (41)	UYL94001.1 hypothetical protein *Geobacillus* phage vB_GthS_PK3.5 (41)	98/97 (41)	2e-18
ORF59 (32,245 -> 32,646)	hypothetical protein (133)	UYL93804.1 hypothetical protein *Geobacillus* phage vB_GthS_NIIg9.7 (135)	66/78 (136)	2e-51
ORF60 (32,663 -> 32,758)	hypothetical protein (31)	No hits found		
ORF61 (32,752 -> 32,835)	hypothetical protein (27)	UYL93852.1 hypothetical protein *Geobacillus* phage vB_GthS_NIIg9.7 (36)	100/100 (17)	3e-07
ORF62 (32,845 -> 33,009)	hypothetical protein (54)	UYL93840.1 hypothetical protein *Geobacillus* phage vB_GthS_NIIg9.7 (58)	98/100 (54)	2e-30
ORF63 (33,029 -> 33,169)	hypothetical protein (46)	UYL93847.1 hypothetical protein *Geobacillus* phage vB_GthS_NIIg9.7 (46)	100/100 (46)	1e-24
ORF64 (33,177 -> 33,383)	hypothetical protein (68)	UYL93832.1 hypothetical protein *Geobacillus* phage vB_GthS_NIIg9.7 (68)	100/100 (68)	4e-39
ORF65 (33,384 -> 33,788)	hypothetical protein (134)	UYL93805.1 hypothetical protein *Geobacillus* phage vB_GthS_NIIg9.7 (134)	95/98 (134)	1e-85
ORF66 (33,788 -> 34,111)	hypothetical protein (107)	UYL93964.1 hypothetical protein *Geobacillus* phage vB_GthS_PK3.5 (108)	91/96 (107)	1e-63
ORF67 (34,108 -> 34,533)	ArpU family transcriptional regulator (141)	UYL93801.1 ArpU family transcriptional regulator *Geobacillus* phage vB_GthS_NIIg9.7 (141)	99/100 (141)	6e-98
ORF68 (35,119 -> 35,886)	DNA methyltransferase (255)	UYL93867.1 DNA methyltransferase *Geobacillus* phage vB_GthS_PK2.1 (286)	65/75 (258)	3e-113
ORF69 (35,910 -> 36,062)	hypothetical protein (50)	UYL93844.1 hypothetical protein *Geobacillus* phage vB_GthS_NIIg9.7 (50)	94/94 (50)	3e-26
ORF70 (36,059 -> 36,814)	metallophosphoesterase (251)	UYL93786.1 metallophosphoesterase *Geobacillus* phage vB_GthS_NIIg9.7 (251)	99/100 (251)	0.0
ORF71 (36,841 -> 37,047)	hypothetical protein (68)	UYL93824.1 hypothetical protein *Geobacillus* phage vB_GthS_NIIg9.7 (79)	100/100 (59)	7e-32
ORF72 (37,096 -> 37,293)	hypothetical protein (65)	UYL93983.1 hypothetical protein *Geobacillus* phage vB_GthS_PK3.5 (65)	95/100 (65)	2e-36
ORF73 (37,308 -> 37,556)	hypothetical protein (82)	UYL93823.1 hypothetical protein *Geobacillus* phage vB_GthS_NIIg9.7 (82)	99/98 (82)	8e-50
ORF74 (37,572 -> 37,967)	HNH endonuclease (131)	UYL93806.1 HNH endonuclease *Geobacillus* phage vB_GthS_NIIg9.7 (131)	97/97 (131)	8e-89
ORF75	hypothetical protein	UYL93816.1 hypothetical protein	72/87 (101)	5e-48

(37,960 -> 38,265)	(101)	*Geobacillus* phage vB_GthS_NIIg9.7 (101)		

Table S5. NIIg9.7 ORFs with homologues in other viruses or cellular organisms.

NIIg-9.7 ORF (position in genome)	Predicted function (protein length (aa))	Significant match (protein length (aa))	Identity/ similarity (%) (length of the overlapping segment (aa))	E value
ORF01<br>(14 -> 508)	terminase small subunit (164)	UYL94219.1 terminase small subunit<br>*Geobacillus* phage vB_GthS_PT9.1 (164)	98/100 (164)	4e-113
ORF02<br>(505 -> 2,208)	terminase large subunit (567)	UYL94201.1 terminase large subunit<br>*Geobacillus* phage vB_GthS_PT9.1 (567)	99/99 (567)	0.0
ORF03<br>(2,213 -> 2,407)	hypothetical protein (64)	UYL94256.1 hypothetical protein<br>*Geobacillus* phage vB_GthS_PT9.1 (64)	92/95 (64)	1e-33
ORF04<br>(2,441 -> 3,673)	portal protein (410)	UYL94205.1 portal protein<br>*Geobacillus* phage vB_GthS_PT9.1 (410)	99/100 (410)	0.0
ORF05<br>(3,673 -> 4,401)	Clp protease (242)	UYL94088.1 Clp protease<br>*Geobacillus* phage vB_GthS_PK5.1 (242)	98/99 (242)	2e-173
ORF06<br>(4,444 -> 5,559)	major capsid protein (371)	UYL93936.1 major capsid protein<br>*Geobacillus* phage vB_GthS_PK3.5 (371)	97/98 (371)	0.0
ORF07<br>(5,605 -> 5,751)	hypothetical protein (48)	YP_001285812.1 hypothetical protein<br>*Geobacillus* virus E2 (48)	98/100 (48)	5e-26
ORF08<br>(5,748 -> 6,035)	head-tail connector protein (95)	UYL94115.1 head-tail connector protein<br>*Geobacillus* phage vB_GthS_PK5.1 (95)	99/98 (94)	5e-61
ORF09<br>(6,032 -> 6,373)	head closure protein (113)	UYL94230.1 head closure protein<br>*Geobacillus* phage vB_GthS_PT9.1 (113)	97/99 (113)	9e-77
ORF10<br>(6,354 -> 6,734)	putative tail-component (126)	UYL94229.1 putative tail component<br>*Geobacillus* phage vB_GthS_PT9.1 (126)	98/100 (126)	3e-85
ORF11<br>(6,736 -> 7,056)	putative tail-component (106)	UYL94234.1 putative tail component<br>*Geobacillus* phage vB_GthS_PT9.1 (106)	98/99 (106)	2e-69
ORF12<br>(7,062 -> 7,640)	major tail protein (192)	UYL94215.1 major tail protein<br>*Geobacillus* phage vB_GthS_PT9. (192)	99/100 (192)	1e-139
ORF13<br>(7,644 -> 7,982)	hypothetical protein (112)	UYL94232.1 hypothetical protein<br>*Geobacillus* phage vB_GthS_PT9.1 (112)	98/100 (112)	9e-75
ORF14<br>(7,933 -> 8,190)	hypothetical protein (85)	UYL94243.1hypothetical protein<br>*Geobacillus* phage vB_GthS_PT9.1 (85)	99/100 (85)	7e-54
ORF15<br>(8,204 -> 10,882)	tape measure protein (892)	UYL94073.1 tape measure protein<br>*Geobacillus* phage vB_GthS_PK5.1 (883)	85/90 (864)	0.0
ORF16<br>(10,895 -> 12,328)	distal tail protein (477)	UYL94202.1 distal tail protein<br>*Geobacillus* phage vB_GthS_PT9.1 (477)	98/99 (477)	0.0
ORF17<br>(12,325 -> 16,038)	tail fiber protein (1237)	UYL94199.1 tail fiber protein<br>*Geobacillus* phage vB_GthS_PT9.1 (1237)	98/98 (1237)	0.0
ORF18<br>(16,035 -> 16,451)	hypothetical protein (138)	YP_009194084.1 hypothetical protein<br>*Brevibacillus* phage Sundance (140)	35/56 (132)	5e-14
ORF19<br>(16,451 -> 16,678)	hypothetical protein (75)	QBP06953.1 hypothetical protein<br>*Virgibacillus* phage Mimir87 (82)	47/79 (68)	4e-18
ORF20<br>(16,716 -> 16,934)	hemolysin XhlA (72)	UYL94248.1 hemolysin<br>*Geobacillus* phage vB_GthS_PT9.1 (72)	99/100 (72)	3e-41
ORF21<br>(16,934 -> 17,194)	holin (86)	UYL94242.1 holin<br>*Geobacillus* phage vB_GthS_PT9.1 (86)	99/100 (86)	3e-50
ORF22<br>(17,194 -> 17,880)	N-acetylmuramoyl-L-alanine amidase (228)	YP_001425618.1 N-acetylmuramoyl-L-alanine amidase<br>Svunavirus sv1 (226)	86/90 (202)	3e-105

ORF23<br>(17,958 -> 18,221)	hypothetical protein (87)	UYL94241.1 hypothetical protein<br>*Geobacillus* phage vB_GthS_PT9.1 (87)	91/93 (87)	2e-50
ORF24<br>(18,375 -> 19,637)	FtsK/SpoIIIE family protein (420)	UYL94204.1 FtsK/SpoIIIE family protein<br>*Geobacillus* phage vB_GthS_PT9.1 (420)	99/98 (420)	0.0
ORF25<br>(19,543 -> 20,160)	replication/relaxation protein (205)	UYL93945.1 replication/relaxation protein<br>*Geobacillus* phage vB_GthS_PK3.5 (205)	98/98 (205)	1e-147
ORF26<br>(20,284 -> 20,472)	XRE family transcriptional regulator (62)	UYL93984.1 XRE family transcriptional regulator<br>*Geobacillus* phage vB_GthS_PK3.5 (62)	98/100 (62)	3e-35
ORF27<br>(20,515 -> 20,721)	hypothetical protein (68)	WP_043904673.1 hypothetical protein<br>*Geobacillus* phage vB_GthS_PT9.1 (68)	99/98 (68)	5e-41
ORF28<br>(20,735 -> 20,968)	hypothetical protein (77)	UYL93973.1 hypothetical protein<br>*Geobacillus* phage vB_GthS_PK3.5 (84)	97/98 (76)	1e-46
ORF29<br>(20,955 -> 21,164)	hypothetical protein (69)	UYL94051.1 hypothetical protein<br>*Geobacillus* phage vB_GthS_PK3.6 (79)	99/100 (68)	1e-38
ORF30<br>(21,169 -> 21,759)	ERF family protein (196)	YP_007236371.1 ERF family protein<br>*Bacillus* phage vB_BtS_BMBtp2 (218)	54/71 (138)	2e-47
ORF31<br>(21,756 -> 21,908)	hypothetical protein (50)	UYL94067.1 hypothetical protein<br>*Geobacillus* phage vB_GthS_PK3.6 (50)	94/96 (50)	3e-24
ORF32<br>(21,896 -> 22,111)	hypothetical protein (71)	UYL94054.1 hypothetical protein<br>*Geobacillus* phage vB_GthS_PK3.6 (71)	99/98 (71)	5e-43
ORF33<br>(22,108 -> 22,221)	hypothetical protein (37)	UYL94002.1 hypothetical protein<br>*Geobacillus* phage vB_GthS_PK3.5 (37)	97/97 (37)	9e-16
ORF34<br>(22,218 -> 22,559)	hypothetical protein (113)	UYL94107.1 hypothetical protein<br>*Geobacillus* phage vB_GthS_PK5.1 (111)	77/79 (117)	5e-45
ORF35<br>(22,552 -> 22,710)	hypothetical protein (52)	WP_098417325.1 hypothetical protein<br>*Bacillus cereus* (90)	61/73 (38)	3e-07
ORF36<br>(22,728 -> 23,108)	hypothetical protein (126)	UYL94228.1 hypothetical protein<br>*Geobacillus* phage vB_GthS_PT9.1 (126)	99/99 (126)	1e-84
ORF37<br>(23,105 -> 23,548)	hypothetical protein (147)	UYL93953.1 hypothetical protein<br>*Geobacillus* phage vB_GthS_PK3.5 (147)	94/97 (147)	3e-97
ORF38<br>(23,545 -> 24,234)	thymidylate synthase (229)	UYL94089.1 thymidylate synthase<br>*Geobacillus* phage vB_GthS_PK5.1 (229)	99/98 (229)	7e-167
ORF39<br>(24,260 -> 24,592)	hypothetical protein (110)	UYL94108.1 hypothetical protein<br>*Geobacillus* phage vB_GthS_PK5.1 (110)	97/99 (110)	3e-72
ORF40<br>(24,965 <- 25,153)	ribbon-helix-helix domain-containing protein (62)	YP_009273374.1 ribbon-helix-helix domain-containing protein<br>*Bacillus* phage vB_BhaS-171 (56)	72/86 (50)	1e-20
ORF41<br>(25,346 -> 25,513)	hypothetical protein (55)	No hits found		
ORF42<br>(25,528 -> 26,277)	Rha family transcriptional regulator (249)	UYL94087.1 Rha family transcriptional regulator<br>*Geobacillus* phage vB_GthS_PK5.1 (242)	88/90 (241)	6e-148
ORF43<br>(26,252 -> 26,548)	hypothetical protein (98)	UYL94114.1 hypothetical protein<br>*Geobacillus* phage vB_GthS_PK5.1 (100)	96/100 (98)	4e-63
ORF44<br>(26,833 -> 27,063)	XRE-family transcriptional regulator (76)	UYL93977.1 XRE-family transcriptional regulator<br>*Geobacillus* phage vB_GthS_PK3.5 (76)	97/100 (75)	2e-44
ORF45<br>(27,143 -> 27,691)	putative endonuclease (182)	WP_236934102.1 DUF559 domain-containing protein<br>*Geobacillus thermodenitrificans* (184)	64/78 (183)	1e-70
ORF46<br>(27,733 -> 28,575)	DNA replication initiation protein DnaD (280)	UYL94207.1 DNA replication initiation protein<br>*Geobacillus* phage vB_GthS_PT9.1 (323)	47/60 (292)	1e-61

ORF47<br>(28,568 -> 28,855)	loader and inhibitor of replicative helicase (95)	QEG13496.1 helicase loader<br>*Bacillus* phage vB_BspS_SplendidRed (123)	40/57 (68)	2e-10
ORF48<br>(28,839 -> 30,110)	replicative DNA helicase (423)	UYL94203.1 replicative DNA helicase<br>*Geobacillus* phage vB_GthS_PT9.1 (423)	99/99 (423)	0.0
ORF49<br>(30,107 -> 30,565)	hypothetical protein (152)	UYL94220.1hypothetical protein<br>*Geobacillus* phage vB_GthS_PT9.1 (152)	97/98 (152)	2e-102
ORF50<br>(30,580 -> 31,116)	HNH endonuclease (178)	UYL94218.1 HNH endonuclease<br>*Geobacillus* phage vB_GthS_PT9.1 (168)	86/91 (168)	9e-90
ORF51<br>(31,140 -> 31,262)	hypothetical protein (40)	UYL94269.1 hypothetical protein<br>*Geobacillus* phage vB_GthS_PT9.1 (40)	98/100 (40)	3e-17
ORF52<br>(31,312 -> 31,731)	single-stranded DNA binding protein (139)	UYL94100.1 single-stranded DNA binding protein<br>*Geobacillus* phage vB_GthS_PK5.1 (141)	96/97 (139)	2e-93
ORF53<br>(31,746 -> 31,943)	hypothetical protein (65)	YP_009010523.1 hypothetical protein<br>*Geobacillus* phage GBK2 (65)	94/100 (65)	7e-28
ORF54<br>(31,961 -> 32,401)	DNA N-6-adenine-methyltransferase (146)	UYL94221.1 DNA N-6-adenine-methyltransferase<br>*Geobacillus* phage vB_GthS_PT9.1 (146)	99/100 (146)	9e-106
ORF55<br>(32,398 -> 32,511)	hypothetical protein (37)	UYL94271.1 hypothetical protein<br>*Geobacillus* phage vB_GthS_PT9.1 (37)	97/100 (37)	3e-15
ORF56<br>(32,508 -> 32,699)	hypothetical protein (63)	UYL94259.1 hypothetical protein<br>*Geobacillus* phage vB_GthS_PT9.1 (60)	100/100 (60)	6e-36
ORF57<br>(32,692 -> 32,877)	hypothetical protein (61)	UYL94128.1 hypothetical protein<br>*Geobacillus* phage vB_GthS_PK5.1 (61)	92/91 (61)	8e-28
ORF58<br>(32,891 -> 33,196)	hypothetical protein (101)	UYL94237.1 hypothetical protein<br>*Geobacillus* phage vB_GthS_PT9.1 (101)	95/97 (101)	2e-65
ORF59<br>(33,174 -> 33,653)	dUTP diphosphatase (159)	YP_008240342.1 dUTP diphosphatase<br>*Thermus* phage phi OH2 (169)	52/69 (171)	4e-47
ORF60<br>(33,663 -> 33,827)	hypothetical protein (54)	YP_007010964.1 hypothetical protein<br>Deep-sea thermophilic phage D6E (59)	96/100 (53)	5e-28
ORF61<br>(33,837 -> 34,148)	holliday junction resolvase (109)	UYL94235.1 holliday junction resolvase<br>*Geobacillus* phage vB_GthS_PT9.1 (103)	98/99 (103)	1e-64
ORF62<br>(34,132 -> 34,257)	hypothetical protein (41)	UYL94001.1 hypothetical protein<br>*Geobacillus* phage vB_GthS_PK3.5 (41)	98/97 (41)	2e-18
ORF63<br>(34,250 -> 34,657)	hypothetical protein (135)	UYL94101.1hypothetical protein<br>*Geobacillus* phage vB_GthS_PK5.1 (135)	93/95 (135)	7e-86
ORF64<br>(34,735 -> 34,845)	hypothetical protein (36)	UYL94273.1 hypothetical protein<br>*Geobacillus* phage vB_GthS_PT9.1 (27)	70/77 (27)	0.019
ORF65<br>(34,845 -> 35,021)	hypothetical protein (58)	UYL94262.1 hypothetical protein<br>*Geobacillus* phage vB_GthS_PT9.1 (54)	98/100 (54)	2e-30
ORF66<br>(35,041 -> 35,181)	hypothetical protein (46)	UYL93998.1 hypothetical protein<br>*Geobacillus* phage vB_GthS_PK3.5 (46)	98/97 (46)	2e-23
ORF67<br>(35,188 -> 35,394)	hypothetical protein (68)	No hits found		
ORF68<br>(35,395 -> 35,799)	hypothetical protein (134)	UYL94225.1 hypothetical protein<br>*Geobacillus* phage vB_GthS_PT9.1 (134)	95/98 (135)	1e-85
ORF69<br>(35,819 -> 36,046)	hypothetical protein (75)	UYL94049.1 hypothetical protein<br>*Geobacillus* phage vB_GthS_PK3.6 (81)	95/97 (75)	7e-45
ORF70<br>(36,051 -> 36,476)	ArpU family transcriptional regulator (141)	UYL94222.1 ArpU family transcriptional regulator<br>*Geobacillus* phage vB_GthS_PT9.1 (141)	99/100 (141)	6e-98
ORF71<br>(36,763 -> 36,915)	hypothetical protein (50)	UYL94264.1 hypothetical protein<br>*Geobacillus* phage vB_GthS_PT9.1 (50)	94/94 (50)	3e-26
ORF72<br>(36,912 -> 37,667)	metallophosphoesterase (251)	UYL94209.1 metallophosphoesterase<br>*Geobacillus* phage vB_GthS_PT9.1 (252)	99/100 (251)	0.0

ORF73<br>(37,694 -> 37,933)	hypothetical protein (79)	UYL94252.1 hypothetical protein<br>*Geobacillus* phage vB_GthS_PT9.1 (68)	100/100 (59)	8e-32
ORF74<br>(37,948 -> 38,196)	hypothetical protein (82)	UYL94244.1 hypothetical protein<br>*Geobacillus* phage vB_GthS_PT9.1 (82)	99/98 (82)	8e-50
ORF75<br>(38,212 -> 38,607)	HNH endonuclease (131)	UYL94227.1 HNH endonuclease<br>*Geobacillus* phage vB_GthS_PT9.1 (131)	97/97 (131)	8e-89
ORF76<br>(38,600 -> 38,905)	hypothetical protein (101)	UYL94238.1 hypothetical protein<br>*Geobacillus* phage vB_GthS_PT9.1 (101)	72/87 (101)	5e-48

Table S6. PK5.1 ORFs with homologues in other viruses or cellular organisms.

PK5.1 ORF (position in genome)	Predicted function (protein length (aa))	Significant match (protein length (aa))	Identity/ similarity (%) (length of the overlapping segment (aa))	E value
ORF01<br>(19 -> 519)	terminase small subunit (166)	YP_001285807.1 putative terminase small subunit<br>*Geobacillus* virus E2 (204)	77/83 (155)	1e-109
ORF02<br>(516 -> 2,219)	terminase large subunit (567)	YP_001285808.1 terminase large subunit<br>*Geobacillus* virus E2 (567)	87/94 (567)	0.0
ORF03<br>(2,233 -> 3,468)	portal protein (411)	UYL93935.1 portal protein<br>*Geobacillus* phage vB_GthS_PK3.5 (411)	99/100 (411)	0.0
ORF04<br>(3,468 -> 4,196)	Clp protease (242)	YP_001285810.1 Clp protease ClpP<br>*Geobacillus* virus E2 (243)	99/99 (242)	0.0
ORF05<br>(4,238 -> 5,353)	major capsid protein (371)	UYL93936.1 major capsid protein<br>*Geobacillus* phage vB_GthS_PK3.5 (371)	97/98 (371)	0.0
ORF06<br>(5,399 -> 5,545)	hypothetical protein (48)	YP_001285812.1 hypothetical protein<br>*Geobacillus* virus E2 (48)	98/100 (48)	5e-26
ORF07<br>(5,542 -> 5,829)	head-tail connector protein (95)	UYL94240.1 head-tail connector protein<br>*Geobacillus* phage vB_GthS_PT9.1 (95)	99/98 (95)	1e-61
ORF08<br>(5,826 -> 6,155)	head closure protein (109)	UYL94230.1 head closure protein<br>*Geobacillus* phage vB_GthS_PT9.1 (113)	100/100 (109)	3e-75
ORF09<br>(6,148 -> 6,528)	putative tail-component (126)	UYL94229.1 putative tail-component<br>*Geobacillus* phage vB_GthS_PT9.1 (126)	98/99 (126)	1e-85
ORF10<br>(6,530 -> 6,850)	putative tail-component (106)	UYL93963.1 putative tail-component<br>*Geobacillus* phage vB_GthS_PK3.5 (109)	100/100 (106)	5e-69
ORF11<br>(6,852 -> 7,433)	major tail protein (193)	UYL93946.1 major tail protein<br>*Geobacillus* phage vB_GthS_PK3.5 (195)	97/98 (191)	2e-135
ORF12<br>(7,437 -> 7,778)	hypothetical protein (113)	UYL93961.1 hypothetical protein<br>*Geobacillus* phage vB_GthS_PK3.5 (113)	92/97 (113)	3e-71
ORF13<br>(7,825 -> 7,986)	hypothetical protein (53)	UYL93971.1 hypothetical protein<br>*Geobacillus* phage vB_GthS_PK3.5 (86)	98/100 (53)	3e-29
ORF14<br>(8,000 -> 10,651)	tape measure protein (883)	UYL93778.1 tape measure protein<br>*Geobacillus* phage vB_GthS_NIIg9.7 (892)	85/90 (864)	0.0
ORF15<br>(10,648 -> 11,457)	distal tail protein (269)	YP_001285822.1 tail family protein<br>*Geobacillus* virus E2 (268)	81/89 (268)	2e-162
ORF16<br>(11,471 -> 13,141)	structural protein (556)	YP_001285824.1 putative pectin lyase<br>*Geobacillus* virus E2 (723)	68/79 (34)	2e-11
ORF17<br>(13,220 -> 15,505)	putative tail fiber protein (761)	WCS66564.1 tail end and central needle protein<br>*Bacillus* phage 0105phi7-2 (804)	50/65 (433)	9e-123
ORF18<br>(15,542 -> 15,760)	hemolysin XhlA (72)	UYL93829.1 hemolysin<br>*Geobacillus* phage vB_GthS_NIIg9.7 (72)	96/97 (72)	2e-39
ORF19<br>(15,757 -> 16,020)	holin (87)	UYL94242.1 holin<br>*Geobacillus* phage vB_GthS_PT9.1 (86)	95/96 (86)	8e-48

ORF20 (16,066 -> 16,563)	HNH endonuclease (165)	YP_009302534.1 HNH-type endonuclease *Bacillus* phage SP-15 (230)	3747/ (115)	5e-09
ORF21 (16,656 -> 17,264)	N-acetylmuramoyl-L-alanine amidase (202)	UYL93943.1 N-acetylmuramoyl-L-alanine amidase *Geobacillus* phage vB_GthS_PK3.5 (228)	97/99 (202)	2e-139
ORF22 (17,343 -> 17,606)	hypothetical protein (87)	UYL93966.1 hypothetical protein *Geobacillus* phage vB_GthS_PK3.5 (95)	86/95 (87)	4e-50
ORF23 (17,760 -> 19,022)	FtsK/SpoIIIE family protein (420)	UYL94204.1 FtsK/SpoIIIE family protein *Geobacillus* phage vB_GthS_PT9.1 (420)	97/98 (419)	0.0
ORF24 (18,928 -> 19,545)	replication/relaxation protein (205)	UYL93945.1 replication/relaxation protein *Geobacillus* phage vB_GthS_PK3.5 (205)	98/99 (205)	3e-147
ORF25 (19,668 -> 19,856)	XRE family transcriptional regulator (62)	UYL93837.1 XRE family transcriptional regulator *Geobacillus* phage vB_GthS_NIIg9.7 (62)	100/100 (62)	1e-35
ORF26 (19,898 -> 20,104)	hypothetical protein (68)	UYL94055.1 hypothetical protein *Geobacillus* phage vB_GthS_PK3.6 (68)	100/100 (68)	4e-41
ORF27 (20,117 -> 20,371)	hypothetical protein (84)	UYL93973.1 hypothetical protein *Geobacillus* phage vB_GthS_PK3.5 (84)	90/96 (84)	4e-49
ORF28 (20,331 -> 20,576)	hypothetical protein (81)	UYL94051.1 hypothetical protein *Geobacillus* phage vB_GthS_PK3.6 (79)	97/100 (79)	1e-47
ORF29 (20,660 -> 21,325)	ERF family protein (221)	UYL94020.1 ERF family protein *Geobacillus* phage vB_GthS_PK3.6 (220)	99/99 (221)	3e-158
ORF30 (21,322 -> 21,474)	hypothetical protein (50)	BBW99044.1 hypothetical protein *Geobacillus* phage vB_GthS_NIIg9.7 (50)	100/100 (50)	3e-26
ORF31 (21,462 -> 21,683)	hypothetical protein (73)	UYL94249.1 hypothetical protein *Geobacillus* phage vB_GthS_PT9.1 (71)	99/98 (69)	4e-42
ORF32 (21,673 -> 22,008)	hypothetical protein (111)	UYL93810.1 hypothetical protein *Geobacillus* phage vB_GthS_NIIg9.7 (113)	77/79 (117)	5e-45
ORF33 (22,001 -> 22,339)	hypothetical protein (112)	WP_043904680.1 hypothetical protein *Parageobacillus* genomosp. 1 (113)	65/77 (94)	3e-26
ORF34 (22,336 -> 22,779)	hypothetical protein (147)	UYL93953.1 hypothetical protein *Geobacillus* phage vB_GthS_PK3.5 (147)	98/100 (147)	4e-101
ORF35 (22,776 -> 23,465)	thymidylate synthase (229)	UYL93789.1 thymidylate synthase *Geobacillus* phage vB_GthS_NIIg9.7 (229)	99/98 (229)	7e-167
ORF36 (23,491 -> 23,823)	hypothetical protein (110)	UYL93812.1 hypothetical protein *Geobacillus* phage vB_GthS_NIIg9.7 (110)	97/99 (110)	3e-72
ORF37 (24,422 -> 25,150)	Rha family transcriptional regulator (242)	UYL93787.1 Rha family transcriptional regulator *Geobacillus* phage vB_GthS_NIIg9.7 (249)	88/99 (241)	6e-148
ORF38 (25,147 -> 25,449)	hypothetical protein (100)	UYL93817.1 hypothetical protein *Geobacillus* phage vB_GthS_NIIg9.7 (98)	96/100 (98)	4e-63
ORF39 (25,545 -> 26,390)	DNA replication initiation protein (281)	UYL93937.1 DNA replication initiation protein *Geobacillus* phage vB_GthS_PK3.5 (281)	100/100 (281)	0.0
ORF40 (26,404 -> 27,699)	replicative DNA helicase (431)	UYL93933.1 replicative DNA helicase *Geobacillus* phage vB_GthS_PK3.5 (431)	100/100 (431)	0.0
ORF41 (27,696 -> 27,956)	hypothetical protein (86)	UYL93972.1 hypothetical protein *Geobacillus* phage vB_GthS_PK3.5 (86)	100/100 (86)	2e-54
ORF42 (27,940 -> 28,461)	HNH endonuclease (173)	UYL93948.1 HNH endonuclease *Geobacillus* phage vB_GthS_PK3.5 (173)	100/100 (173)	8e-125
ORF43 (28,678 -> 29,478)	phosphoadenosine phosphosulphate reductase (266)	QPZ53446.1 putative phosphoadenosine phosphosulfate reductase *Pelagibacter* phage HTVC034P (273)	49/67 (274)	3e-83
ORF44 (29,488 -> 29,913)	single-stranded DNA-binding protein	UYL93802.1 single-stranded DNA-binding protein	96/97 (139)	3e-93

	(141)	*Geobacillus* phage vB_GthS_NIIg9.7 (139)		
ORF45 (29,926 -> 30,192)	putative transcriptional regulator (88)	UYL93969.1 putative transcriptional regulator *Geobacillus* phage vB_GthS_PK3.5 (88)	99/98 (88)	2e-56
ORF46 (30,189 -> 30,380)	hypothetical protein (63)	UYL93836.1 hypothetical protein *Geobacillus* phage vB_GthS_NIIg9.7 (63)	100/100 (63)	2e-38
ORF47 (30,373 -> 30,558)	hypothetical protein (61)	UYL93839.1 hypothetical protein *Geobacillus* phage vB_GthS_NIIg9.7 (61)	92/91 (61)	8e-28
ORF48 (30,572 -> 30,850)	hypothetical protein (92)	UYL93815.1 hypothetical protein *Geobacillus* phage vB_GthS_NIIg9.7 101)	95/98 (92)	4e-59
ORF49 (30,853 -> 31,179)	nucleoside triphosphate pyrophosphohydrolase (108)	YP_009273386.1 MazG nucleotide pyrophosphohydrolase domain protein *Bacillus* phage vB_BhaS-171 (108)	63/76 (106)	2e-35
ORF50 (31,180 -> 31,359)	hypothetical protein (59)	YP_007010964.1 hypothetical protein Deep-sea thermophilic phage D6E (59)	92/94 (59)	3e-30
ORF51 (31,373 -> 31,552)	hypothetical protein (59)	WP_110107634.1 hypothetical protein *Geobacillus thermoleovorans* (59)	86/89 (59)	4e-25
ORF52 (31,549 -> 31,860)	holliday junction resolvase (103)	UYL93814.1 holliday junction resolvase *Geobacillus* phage vB_GthS_NIIg9.7 (103)	97/98 (103)	3e-63
ORF53 (31,844 -> 31,969)	hypothetical protein (41)	UYL94001.1 hypothetical protein *Geobacillus* phage vB_GthS_PK3.5 (41)	96/96 (28)	3e-10
ORF54 (31,962 -> 32,369)	hypothetical protein (135)	UYL93804.1 hypothetical protein *Geobacillus* phage vB_GthS_NIIg9.7 (135)	93/95 (135)	7e-86
ORF55 (32,450 -> 32,590)	hypothetical protein (46)	UYL93998.1 hypothetical protein *Geobacillus* phage vB_GthS_PK3.5 (46)	96/95 (46)	2e-22
ORF56 (32,595 -> 32,999)	hypothetical protein (134)	UYL93957.1 hypothetical protein *Geobacillus* phage vB_GthS_PK3.5 (134)	99/99 (134)	1e-88
ORF57 (32,999 -> 33,322)	hypothetical protein (107)	UYL93964.1 hypothetical protein *Geobacillus* phage vB_GthS_PK3.5 (108)	92/95 (105)	1e-62
ORF58 (33,319 -> 33,759)	RinA family transcriptional regulator (146)	UYL94028.1 RinA family transcriptional regulator *Geobacillus* phage vB_GthS_PK3.6 (147)	78/89 (141)	1e-76
ORF59 (33,868 -> 34,548)	hypothetical protein (226)	QRD99627.1 hypothetical protein *Lactobacillus* phage vB_Lcr_AB1 (60)	59/68 (54)	2e-12
ORF60 (35,161 -> 35,913)	DNA methyltransferase (250)	UYL94208.1 DNA-methyltransferase *Geobacillus* phage vB_GthS_PT9.1 (255)	67/74 (257)	4e-109
ORF61 (35,937 -> 36,077)	hypothetical protein (46)	UYL93974.1 hypothetical protein *Geobacillus* phage vB_GthS_PK3.5 (82)	91/97 (46)	2e-23
ORF62 (36,079 -> 36,879)	DNA methyltransferase (266)	UYL93938.1 DNA methyltransferase *Geobacillus* phage vB_GthS_PK3.5 (273)	63/72 (273)	3e-108
ORF63 (36,876 -> 37,631)	metallophosphoesterase (251)	UYL93786.1 metallophosphoesterase *Geobacillus* phage vB_GthS_NIIg9.7 (251)	96/98 (251)	0.0
ORF64 (37,678 -> 38,070)	HNH endonuclease (130)	UYL94227.1 HNH endonuclease *Geobacillus* phage vB_GthS_PT9.1 (131)	92/97 (130)	4e-85

Table S7. PK3.5 ORFs with homologues in other viruses or cellular organisms.

PK3.5 ORF (position in genome)	Predicted function (protein length (aa))	Significant match (protein length (aa))	Identity/ similarity (%) (length of the overlapping segment (aa))	E value
ORF01 (16 -> 510)	terminase small subunit (164)	UYL94219.1 terminase small subunit *Geobacillus* phage vB_GthS_PT9.1 (164)	99/100 (164)	9e-114
ORF02 (507 -> 2,210)	terminase large subunit (567)	UYL94007.1 terminase large subunit *Geobacillus* phage vB_GthS_PK3.6 (567)	99/99 (567)	0.0
ORF03 (2,224 -> 3,459)	portal protein (411)	UYL94079.1 portal protein *Geobacillus* phage vB_GthS_PK5.1 (411)	99/100 (411)	0.0
ORF04 (3,456 -> 4,193)	Clp protease (245)	UYL94210.1 Clp protease *Geobacillus* phage vB_GthS_PT9.1 (244)	99/100 (244)	4e-177
ORF05 (4,233 -> 5,348)	major capsid protein (371)	UYL94206.1 major capsid protein *Geobacillus* phage vB_GthS_PT9.1 (371)	98/98 (371)	0.0
ORF06 (5,395 -> 5,541)	hypothetical protein (48)	YP_001285812.1 hypothetical protein *Geobacillus* virus E2 (48)	98/100 (48)	4e-26
ORF07 (5,538 -> 5,825)	head-tail connector protein (95)	UYL94043.1 head-tail connector protein *Geobacillus* phage vB_GthS_PK3.6 (95)	99/100 (95)	1e-62
ORF08 (5,822 -> 6,151)	head closure protein (109)	YP_001285814.1 head closure protein *Geobacillus* virus E2 (109)	88/95 (109)	4e-69
ORF09 (6,144 -> 6,524)	putative tail-component (126)	UYL93808.1 putative tail component *Geobacillus* phage vB_GthS_NIIg9.7 (126)	94/96 (126)	5e-84
ORF10 (6,517 -> 6,846)	putative tail-component (109)	UYL94039.1 putative tail-component *Geobacillus* phage vB_GthS_PK3.6 (106)	100/100 (106)	5e-69
ORF11 (6,851 -> 7,438)	major tail protein (195)	UYL94022.1 major tail protein *Geobacillus* phage vB_GthS_PK3.6 (192)	99/99 (189)	2e-136
ORF12 (7,435 -> 7,776)	hypothetical protein (113)	UYL94105.1 hypothetical protein *Geobacillus* phage vB_GthS_PK5.1 (113)	92/97 (113)	3e-71
ORF13 (7,724 -> 7,984)	hypothetical protein (86)	UYL94047.1 hypothetical protein *Geobacillus* phage vB_GthS_PK3.6 (85)	94/96 (86)	1e-49
ORF14 (7,998 -> 10,607)	tape measure protein (869)	UYL94006.1 tape measure protein *Geobacillus* phage vB_GthS_PK3.6 (869)	99/99 (869)	0.0
ORF15 (10,611 -> 12,044)	distal tail protein (477)	UYL94008.1 distal tail protein *Geobacillus* phage vB_GthS_PK3.6 (477)	98/98 (477)	0.0
ORF16 (12,041 -> 14,920)	putative tail fiber protein (959)	UYL94005.1 putative tail fiber protein *Geobacillus* phage vB_GthS_PK3.6 (1307)	85/88 (769)	0.0
ORF17 (14,936 -> 15,238)	hypothetical protein (100)	WP_027410375.1 hypothetical protein *Anoxybacillus tepidamans* (93)	55/74 (100)	6e-23
ORF18 (15,543 -> 15,677)	hypothetical protein (44)	UYL94070.1 hypothetical protein *Geobacillus* phage vB_GthS_PK3.6 (44)	98/100 (44)	3e-22
ORF19 (15,679 -> 15,855)	hypothetical protein (58)	UYL94192.1 hypothetical protein *Geobacillus* phage vB_GthS_PK5.2 (50)	53/72 (55)	1e-07
ORF20 (15,845 -> 16,033)	hypothetical protein (62)	WP_081130604.1 hypothetical protein *Geobacillus thermoleovorans* (62)	92/96 (60)	8e-21
ORF21 (16,049 -> 16,246)	hemolysin (65)	UYL94124.1 hemolysin *Geobacillus* phage vB_GthS_PK5.1 (72)	89/90 (72)	2e-33
ORF22 (16,246 -> 16,506)	holin (86)	YP_007010940.1 holin Deep-sea thermophilic phage D6E (87)	88/95 (86)	4e-39
ORF23 (16,506 -> 17,192)	N-acetylmuramoyl-L-alanine amidase (228)	UYL94018.1 N-acetylmuramoyl-L-alanine amidase *Geobacillus* phage vB_GthS_PK3.6 (228)	97/98 (228)	1e-160

ORF24 (17,224 -> 17,511)	hypothetical protein (95)	UYL94241.1 hypothetical protein *Geobacillus* phage vB_GthS_PT9.1 (87)	90/95 (87)	1e-50
ORF25 (17,665 -> 18,927)	FtsK/SpoIIIE family protein (420)	UYL94010.1 FtsK/SpoIIIE family protein *Geobacillus* phage vB_GthS_PK3.6 (420)	98/98 (420)	0.0
ORF26 (18,833 -> 19,450)	replication/relaxation protein (205)	UYL93791.1 replication/relaxation protein *Geobacillus* phage vB_GthS_NIIg9.7 (205)	98/98 (205)	1e-147
ORF27 (19,575 -> 19,763)	XRE family transcriptional regulator (62)	UYL93837.1 XRE family transcriptional regulator *Geobacillus* phage vB_GthS_NIIg9.7 (62)	98/100 (62)	3e-35
ORF28 (19,805 -> 20,011)	hypothetical protein (68)	UYL94253.1 hypothetical protein *Geobacillus* phage vB_GthS_PT9.1 (68)	99/98 (68)	1e-40
ORF29 (20,024 -> 20,278)	hypothetical protein (84)	UYL94121.1 hypothetical protein *Geobacillus* phage vB_GthS_PK5.1 (84)	90/96 (84)	4e-49
ORF30 (20,244 -> 20,483)	hypothetical protein (79)	UYL94051.1 hypothetical protein *Geobacillus* phage vB_GthS_PK3.6 (79)	96/96 (79)	2e-46
ORF31 (20,567 -> 21,232)	ERF family protein (221)	UYL94091.1 ERF family protein *Geobacillus* phage vB_GthS_PK5.1 (221)	97/98 (221)	2e-156
ORF32 (21,229 -> 21,357)	hypothetical protein (42)	UYL93845.1 hypothetical protein *Geobacillus* phage vB_GthS_NIIg9.7 (50)	100/100 (41)	1e-18
ORF33 (21,345 -> 21,560)	hypothetical protein (71)	UYL93830.1 hypothetical protein *Geobacillus* phage vB_GthS_NIIg9.7 (71)	94/95 (70)	7e-40
ORF34 (21,557 -> 21,670)	hypothetical protein (37)	UYL93850.1 hypothetical protein *Geobacillus* phage vB_GthS_NIIg9.7 (37)	97/97 (37)	9e-16
ORF35 (21,667 -> 21,834)	hypothetical protein (55)	UAW07755.1 hypothetical protein *Psychrobacillus* phage PVJ1 (60)	48/63 (52)	4e-10
ORF36 (21,831 -> 22,208)	hypothetical protein (125)	UYL94036.1 hypothetical protein *Geobacillus* phage vB_GthS_PK3.6 (114)	89/91 (99)	9e-57
ORF37 (22,205 -> 22,648)	hypothetical protein (147)	UYL94098.1 hypothetical protein *Geobacillus phage vB_GthS_PK5.1* (147)	98/100 (147)	3e-101
ORF38 (22,645 -> 23,187)	hypothetical protein (180)	UYL94023.1 hypothetical protein *Geobacillus phage vB_GthS_PK3.6* (180)	96/98 (180)	2e-114
ORF39 (23,776 -> 24,006)	XRE family transcriptional regulator (76)	UYL93826.1 XRE family transcriptional regulator *Geobacillus* phage vB_GthS_NIIg9.7 (76)	97/100 (75)	2e-44
ORF40 (24,088 -> 24,834)	antirepressor protein (248)	ATN94298.1 antirepressor protein *Lysinibacillus* phage vB_LspM-01 (238)	59/71 (215)	1e-81
ORF41 (24,844 -> 24,903)	hypothetical protein (19)	No hits found		
ORF42 (24,922 -> 25,008)	hypothetical protein (28)	No hits found		
ORF43 (25,043 -> 25,264)	XRE family transcriptional regulator (73)	APU92986.1 transcriptional regulator *Ochrobactrum* phage POI1126 (74)	32/57 (69)	1e-06
ORF44 (25,309 -> 25,485)	hypothetical protein (58)	No hits found		
ORF45 (25,548 -> 26,393)	DNA replication initiation protein DnaD (281)	UYL94013.1 DNA replication initiation protein *Geobacillus* phage vB_GthS_PK3.6 (283)	98/98 (283)	0.0
ORF46 (26,407 -> 27,702)	replicative DNA helicase (431)	YP_001285846.1 replicative DNA helicase *Geobacillus* virus E2 (435)	66/82 (430)	0.0
ORF47 (26,407 -> 27,702)	hypothetical protein (86)	YP_001285847.1 hypothetical protein *Geobacillus* virus E2 (69)	76/79 (67)	1e-43
ORF48 (27,943 -> 28,464)	HNH endonuclease (173)	UYL94026.1 HNH endonuclease *Geobacillus* phage vB_GthS_PK3.6 (169)	73/82 (158)	1e-67

ORF49<br>(28,461 -> 28,610)	hypothetical protein<br>(49)	UYL93849.1 hypothetical protein<br>*Geobacillus* phage vB_GthS_NIIg9.7 (40)	88/95 (40)	2e-14
ORF50<br>(28,660 -> 29,088)	single-stranded DNA-binding protein<br>(142)	UYL94030.1 single-stranded DNA-binding protein<br>*Geobacillus* phage vB_GthS_PK3.6 (141)	87/91 (142)	1e-83
ORF51<br>(29,101 -> 29,541)	DNA N-6-adenine-methyltransferase (146)	UYL94221.1 N-6-adenine-methyltransferase<br>*Geobacillus* phage vB_GthS_PT9.1 (146)	99/100 (144)	1e-103
ORF52<br>(29,538 -> 29,804)	putative transcriptional regulator (88)	UYL94117.1 putative transcriptional regulator<br>*Geobacillus* phage vB_GthS_PK5.1 (88)	99/98 (88)	2e-56
ORF53<br>(29,801 -> 29,983)	hypothetical protein<br>(60)	UYL93836.1 hypothetical protein<br>*Geobacillus* phage vB_GthS_NIIg9.7 (63)	93/96 (60)	4e-33
ORF54<br>(29,985 -> 30,170)	hypothetical protein<br>(61)	UYL94258.1 hypothetical protein<br>*Geobacillus* phage vB_GthS_PT9.1 (61)	98/98 (61)	2e-30
ORF55<br>(30,184 -> 30,471)	hypothetical protein<br>(95)	UYL93815.1 hypothetical protein<br>*Geobacillus* phage vB_GthS_NIIg9.7 (101)	95/96 (93)	2e-58
ORF56<br>(30,468 -> 30,656)	hypothetical protein<br>(62)	WP_060476219.1 hypothetical protein<br>*Geobacillus* sp. PA-3 (62)	98/98 (62)	3e-61
ORF57<br>(30,660 -> 31,172)	dUTP diphosphatase<br>(170)	UYL94025.1 dUTP diphosphatase<br>*Geobacillus* phage vB_GthS_PK3.6 (170)	98/100 (170)	3e-119
ORF58<br>(31,186 -> 31,350)	hypothetical protein<br>(54)	YP_001285856.1 hypothetical protein<br>*Geobacillus* virus E2 (54)	89/98 (54)	2e-27
ORF59<br>(31,347 -> 31,514)	hypothetical protein<br>(55)	WP_068894754.1 hypothetical protein<br>*Geobacillus thermoleovorans* (60)	93/95 (44)	2e-36
ORF60<br>(31,554 -> 32,012)	YopX family protein<br>(152)	YP_007010965.1 sensor protein<br>Deep-sea thermophilic phage D6E (143)	56/58 (175)	1e-70
ORF61<br>(32,009 -> 32,164)	hypothetical protein<br>(51)	YP_001285858.1 hypothetical protein<br>*Geobacillus* virus E2 (51)	96/98 (51)	2e-28
ORF62<br>(32,214 -> 32,732)	Holliday junction resolvase (172)	UYL94159.1 holliday junction resolvase<br>*Geobacillus* phage vB_GthS_PK5.2 (165)	88/92 (162)	3e-100
ORF63<br>(32,726 -> 32,851)	hypothetical protein<br>(41)	UYL93848.1 hypothetical protein<br>*Geobacillus* phage vB_GthS_NIIg9.7 (41)	98/97 (41)	2e-18
ORF64<br>(32,913 -> 33,053)	hypothetical protein<br>(46)	UYL94063.1 hypothetical protein<br>*Geobacillus* phage vB_GthS_PK3.6 (58)	98/97 (46)	9e-24
ORF65<br>(33,058 -> 33,462)	hypothetical protein<br>(134)	UYL94102.1 hypothetical protein<br>*Geobacillus* phage vB_GthS_PK5.1 (134)	99/99 (134)	1e-88
ORF66<br>(33,459 -> 33,785)	hypothetical protein<br>(108)	UYL94233.1 hypothetical protein<br>*Geobacillus* phage vB_GthS_PT9.1 (107)	91/96 (107)	1e-63
ORF67<br>(33,782 -> 34,219)	RinA family transcriptional regulator<br>(145)	UYL94028.1 RinA family transcriptional regulator<br>*Geobacillus* phage vB_GthS_PK3.6 (147)	86/93 (143)	2e-85
ORF68<br>(34,222 -> 34,386)	hypothetical protein<br>(54)	No hits found		
ORF69<br>(35,006 -> 35,734)	DNA methyltransferase<br>(242)	APC42946.1 adenine-specific methyltransferase<br>*Streptococcus* phage IPP34 (239)	37/61 (239)	6e-44
ORF70<br>(35,772 -> 35,990)	hypothetical protein<br>(72)	No hits found		
ORF71<br>(35,994 -> 36,242)	hypothetical protein<br>(82)	UYL94264.1 hypothetical protein<br>*Geobacillus* phage vB_GthS_PT9.1 (50)	85/91 (48)	2e-22
ORF72<br>(36,239 -> 37,060)	DNA methyltransferase<br>(273)	UYL94084.1 DNA methyltransferase<br>*Geobacillus* phage vB_GthS_PK5.1 (266)	63/72 (273)	3e-108
ORF73<br>(37,017 -> 37,799)	metallophosphoesterase<br>(260)	UYL94014.1 metallophosphoesterase<br>*Geobacillus* phage vB_GthS_PK3.6 (252)	97/99 (252)	0.0
ORF74<br>(37,849 -> 38,046)	hypothetical protein<br>(65)	UYL94255.1 hypothetical protein<br>*Geobacillus* phage vB_GthS_PT9.1 (65)	95/100 (65)	2e-36

ORF75 (38,061 -> 38,303)	hypothetical protein (80)	UYL93823.1 hypothetical protein *Geobacillus* phage vB_GthS_NIIg9.7 (82)	68/82 (76)	2e-29
ORF76 (38,303 -> 38,695)	HNH endonuclease (130)	UYL94103.1 HNH endonuclease *Geobacillus* phage vB_GthS_PK5.1 (130)	87/94 (130)	3e-80

Table S8. PK3.6 ORFs with homologues in other viruses or cellular organisms.

PK3.6 ORF (position in genome)	Predicted function (protein length (aa))	Significant match (protein length (aa))	Identity/ similarity (%) (length of the overlapping segment (aa))	E value
ORF01 (16 -> 510)	terminase small subunit (164)	UYL93951.1 terminase small subunit *Geobacillus* phage vB_GthS_PK3.5 (164)	100/100 (164)	1e-114
ORF02 (507 -> 2,210)	terminase large subunit (567)	UYL93931.1 terminase large subunit *Geobacillus* phage vB_GthS_PK3.5 (567)	99/99 (567)	0.0
ORF03 (2,224 -> 3,459)	portal protein (411)	UYL93935.1 portal protein *Geobacillus* phage vB_GthS_PK3.5 (411)	100/100 (411)	0.0
ORF04 (3,456 -> 4,193)	Clp protease (245)	UYL93941.1 Clp protease *Geobacillus* phage vB_GthS_PK3.5 (245)	100/100 (232)	3e-179
ORF05 (4,233 -> 5,348)	major capsid protein (371)	UYL93936.1 major capsid protein *Geobacillus* phage vB_GthS_PK3.5 (371)	100/100 (371)	0.0
ORF06 (5,394 -> 5,540)	hypothetical protein (48)	UYL93846.1 hypothetical protein *Geobacillus* phage vB_GthS_NIIg9.7 (48)	100/100 (48)	5e-24
ORF07 (5,537 -> 5,824)	head-tail connector protein (95)	UYL93967.1 head-tail connector protein *Geobacillus* phage vB_GthS_PK3.5 (95)	99/100 (95)	1e-62
ORF08 (5,821 -> 6,150)	head closure protein (109)	UYL93962.1 head closure protein *Geobacillus* phage vB_GthS_PK3.5 (109)	100/100 (109)	9e-75
ORF09 (6,143 -> 6,523)	putative tail-component (126)	UYL93959.1 putative tail-component *Geobacillus* phage vB_GthS_PK3.5 (126)	84/92 (126)	6e-86
ORF10 (6,525 -> 6,845)	putative tail-component (106)	UYL93963.1 putative tail-component *Geobacillus* phage vB_GthS_PK3.5 (109)	100/100 (106)	5e-69
ORF11 (6,850 -> 7,428)	major tail protein (192)	UYL93946.1 major tail protein *Geobacillus* phage vB_GthS_PK3.5 (195)	99/99 (189)	2e-136
ORF12 (7,432 -> 7,770)	hypothetical protein (112)	UYL94232.1 hypothetical protein *Geobacillus* phage vB_GthS_PT9.1 (112)	97/100 (112)	2e-74
ORF13 (7,721 -> 7,978)	hypothetical protein (85)	UYL94243.1 hypothetical protein *Geobacillus* phage vB_GthS_PT9.1 (85)	94/96 (85)	1e-50
ORF14 (7,991 -> 10,600)	tape measure protein (869)	UYL93930.1 tape measure protein *Geobacillus* phage vB_GthS_PK3.5 (869)	99/99 (869)	0.0
ORF15 (10,604 -> 12,037)	distal tail protein (433)	UYL93932.1 distal tail protein *Geobacillus* phage vB_GthS_PK3.5 (477)	98/98 (477)	0.0
ORF16 (12,028 -> 15,951)	putative tail fiber protein (1307)	UYL93929.1 putative tail fiber protein *Geobacillus* phage vB_GthS_PK3.5 (959)	86/89 (769)	0.0
ORF17 (15,967 -> 16,269)	hypothetical protein (100)	QAX97583.1 hypothetical protein *Enterococcus* phage EfsSzw-1 (96)	30/51 (102)	1e-05
ORF18 (16,575 -> 16,709)	hypothetical protein (44)	UYL93999.1 hypothetical protein *Geobacillus* phage vB_GthS_PK3.5 (44)	98/100 (44)	3e-22
ORF19 (16,711 -> 16,887)	hypothetical protein (58)	UYL93989.1 hypothetical protein *Geobacillus* phage vB_GthS_PK3.5 (58)	100/100 (58)	1e-29
ORF20 (16,877 -> 17,065)	hypothetical protein (62)	UYL93985.1 hypothetical protein *Geobacillus* phage vB_GthS_PK3.5 (62)	100/100 (62)	2e-35
ORF21 (17,081 -> 17,278)	hemolysin XhlA (65)	UYL93982.1 hemolysin *Geobacillus* phage vB_GthS_PK3.5 (65)	100/100 (65)	6e-37

ORF22 (17,278 -> 17,538)	holin (86)	UYL93970.1 holin *Geobacillus* phage vB_GthS_PK3.5 (86)	100/100 (86)	2e-51
ORF23 (17,538 -> 18,224)	N-acetylmuramoyl-L-alanine amidase (228)	UYL93943.1 N-acetylmuramoyl-L-alanine amidase *Geobacillus* phage vB_GthS_PK3.5 (228)	97/98 (228)	1e-160
ORF24 (18,350 -> 18,565)	hypothetical protein (71)	UYL94119.1 hypothetical protein *Geobacillus* phage vB_GthS_PK5.1 (87)	93/97 (71)	4e-41
ORF25 (18,720 -> 19,982)	FtsK/SpoIIIE family protein (420)	UYL93934.1 FtsK/SpoIIIE family protein *Geobacillus* phage vB_GthS_PK3.5 (420)	98/98 (420)	0.0
ORF26 (19,888 -> 20,505)	replication/relaxation protein (205)	UYL93945.1 replication/relaxation protein *Geobacillus* phage vB_GthS_PK3.5 (205)	97/99 (205)	2e-146
ORF27 (20,631 -> 20,819)	XRE family transcriptional regulator (62)	UYL93837.1 XRE family transcriptional regulator *Geobacillus* phage vB_GthS_NIIg9.7 (62)	97/100 (62)	1e-34
ORF28 (20,869 -> 21,075)	hypothetical protein (68)	UYL94253.1 hypothetical protein *Geobacillus* phage vB_GthS_PT9.1 (68)	74/83 (68)	5e-29
ORF29 (21,089 -> 21,343)	hypothetical protein (84)	UYL94121.1 hypothetical protein *Geobacillus* phage vB_GthS_PK5.1 (84)	87/94 (84)	3e-46
ORF30 (21,309 -> 21,548)	hypothetical protein (79)	UYL94122.1 hypothetical protein *Geobacillus* phage vB_GthS_PK5.1 (81)	97/100 (79)	1e-47
ORF31 (21,632 -> 22,294)	ERF family protein (220)	UYL94091.1 ERF family protein *Geobacillus* phage vB_GthS_PK5.1 (221)	99/99 (221)	3e-158
ORF32 (22,291 -> 22,443)	hypothetical protein (50)	UYL93845.1 hypothetical protein *Geobacillus* phage vB_GthS_NIIg9.7 (50)	94/96 (50)	3e-24
ORF33 (22,431 -> 22,646)	hypothetical protein (71)	UYL93830.1 hypothetical protein *Geobacillus* phage vB_GthS_NIIg9.7 (71)	99/98 (71)	5e-43
ORF34 (22,643 -> 22,756)	hypothetical protein (37)	UYL94002.1 hypothetical protein *Geobacillus* phage vB_GthS_PK3.5 (37)	92/94 (37)	3e-15
ORF35 (22,753 -> 22,920)	hypothetical protein (55)	UAW07755.1 hypothetical protein *Psychrobacillus* phage PVJ1 (60)	48/63 (52)	4e-10
ORF36 (22,917 -> 23,261)	hypothetical protein (114)	UYL93960.1 hypothetical protein *Geobacillus* phage vB_GthS_PK3.5 (125)	89/91 (99)	8e-57
ORF37 (23,258 -> 23,566)	hypothetical protein (102)	UYL94236.1 hypothetical protein *Geobacillus* phage vB_GthS_PT9.1 (102)	97/100 (102)	1e-66
ORF38 (23,563 -> 24,105)	hypothetical protein (180)	UYL94216.1 hypothetical protein *Geobacillus* phage vB_GthS_PT9.1 (180)	96/98 (180)	2e-115
ORF39 (24,529 -> 24,759)	XRE family transcriptional regulator (76)	UYL93826.1 XRE family transcriptional regulator *Geobacillus* phage vB_GthS_NIIg9.7 (76)	97/100 (75)	2e-44
ORF40 (25,092 -> 25,943)	DNA replication initiation protein (283)	UYL93937.1 DNA replication initiation protein *Geobacillus* phage vB_GthS_PK3.5 (281)	98/98 (283)	0.0
ORF41 (25,957 -> 27,252)	replicative DNA helicase (431)	UYL93933.1 replicative DNA helicase *Geobacillus* phage vB_GthS_PK3.5 (431)	100/100 (430)	0.0
ORF42 (27,249 -> 27,509)	hypothetical protein (86)	UYL93972.1 hypothetical protein *Geobacillus* phage vB_GthS_PK3.5 (86)	100/100 (86)	2e-54
ORF43 (27,493 -> 28,002)	HNH endonuclease (169)	YP_001285849.1 HNH endonuclease *Geobacillus* virus E2 (188)	72/81 (169)	2e-77
ORF44 (27,944 -> 28,675)	hypothetical protein (243)	QEG13500.1 putative DNA modification protein *Bacillus* phage vB_BspS_SplendidRed (198)	73/89 (191)	1e-106
ORF45 (28,780 -> 29,205)	single-stranded DNA-binding protein (141)	UYL93956.1 single-stranded DNA-binding protein *Geobacillus* phage vB_GthS_PK3.5 (142)	87/91 (142)	1e-83
ORF46 (29,218 -> 29,658)	DNA N-6-adenine-methyltransferase (146)	UYL93954.1 DNA N-6-adenine-methyltransferase *Geobacillus* phage vB_GthS_PK3.5 (146)	100/100 (146)	2e-106

ORF47<br>(29,655 -> 29,921)	putative transcriptional regulator (88)	UYL93969.1 putative transcriptional regulator<br>*Geobacillus* phage vB_GthS_PK3.5 (88)	100/100 (88)	4e-57
ORF48<br>(29,918 -> 30,100)	hypothetical protein (60)	UYL93988.1 hypothetical protein<br>*Geobacillus* phage vB_GthS_PK3.5 (60)	100/100 (60)	9e-36
ORF49<br>(30,102 -> 30,287)	hypothetical protein (61)	UYL93987.1 hypothetical protein<br>*Geobacillus* phage vB_GthS_PK3.5 (61)	100/100 (61)	2e-31
ORF50<br>(30,301 -> 30,588)	hypothetical protein (95)	UYL93968.1 hypothetical protein<br>*Geobacillus* phage vB_GthS_PK3.5 (95)	100/100 (95)	6e-64
ORF51<br>(30,585 -> 30,773)	hypothetical protein (62)	UYL93986.1 hypothetical protein<br>*Geobacillus* phage vB_GthS_PK3.5 (62)	100/100 (62)	5e-37
ORF52<br>(30,777 -> 31,289)	dUTP diphosphatase (170)	UYL93950.1 dUTP diphosphatase<br>*Geobacillus* phage vB_GthS_PK3.5 (170)	98/100 (170)	3e-119
ORF53<br>(31,303 -> 31,467)	hypothetical protein (54)	UYL94130.1 hypothetical protein<br>*Geobacillus* phage vB_GthS_PK5.1 (59)	94/100 (54)	2e-26
ORF54<br>(31,477 -> 31,887)	Holliday junction resolvase (136)	YP_009837571.1 Holliday junction resolvase<br>*Anoxybacillus* phage A403 (137)	71/83 (136)	1e-60
ORF55<br>(31,930 -> 32,106)	hypothetical protein (58)	UYL93998.1 hypothetical protein<br>*Geobacillus* phage vB_GthS_PK3.5 (46)	98/97 (46)	1e-23
ORF56<br>(32,111 -> 32,515)	hypothetical protein (134)	UYL93957.1 hypothetical protein<br>*Geobacillus* phage vB_GthS_PK3.5 (134)	99/99 (134)	2e-88
ORF57<br>(32,517 -> 32,762)	hypothetical protein (81)	UYL93828.1 hypothetical protein<br>*Geobacillus* phage vB_GthS_NIIg9.7 (75)	95/97 (75)	7e-45
ORF58<br>(32,875 -> 33,396)	HNH endonuclease (173)	YP_009837559.1 terminase small subunit<br>*Anoxybacillus* phage A403 (181)	46/65 (163)	4e-40
ORF59<br>(33,359 -> 33,802)	RinA family transcriptional regulator (147)	UYL93955.1 RinA family transcriptional regulator<br>*Geobacillus* phage vB_GthS_PK3.5 (145)	86/93 (143)	2e-85
ORF60<br>(34,457 -> 34,849)	hypothetical protein (130)	QIG62606.1 hypothetical protein<br>*Sporosarcina* phage Lietuvens (134)	49/67 (49)	1e-04
ORF61<br>(34,939 -> 35,661)	DNA methyltransferase (240)	YP_009904999.1 DNA methyltransferase<br>*Lactococcus* phage AM1 (227)	51/69 (227)	2e-77
ORF62<br>(35,685 -> 35,825)	hypothetical protein (46)	UYL93844.1 hypothetical protein<br>*Geobacillus* phage vB_GthS_NIIg9.7 (50)	93/95 (44)	1e-20
ORF63<br>(35,900 -> 36,019)	DNA methyltransferase (39)	UYL93938.1 DNA methyltransferase<br>*Geobacillus* phage vB_GthS_PK3.5 (273)	81/93 (31)	4e-10
ORF64<br>(36,012 -> 36,677)	DNA methyltransferase (221)	UYL94084.1 DNA methyltransferase<br>*Geobacillus* phage vB_GthS_PK5.1 (266)	69/77 (213)	4e-97
ORF65<br>(36,658 -> 37,416)	metallophosphoesterase (252)	UYL93939.1 metallophosphoesterase<br>*Geobacillus* phage vB_GthS_PK3.5 (260)	97/99 (252)	0.0
ORF66<br>(37,466 -> 37,663)	hypothetical protein (65)	UYL93983.1 hypothetical protein<br>*Geobacillus* phage vB_GthS_PK3.5 (65)	100/100 (65)	7e-38
ORF67<br>(37,678 -> 37,920)	hypothetical protein (80)	UYL93975.1 hypothetical protein<br>*Geobacillus* phage vB_GthS_PK3.5 (80)	100/100 (80)	8e-48
ORF68<br>(37,920 -> 38,312)	HNH endonuclease (130)	UYL93958.1 HNH endonuclease<br>*Geobacillus* phage vB_GthS_PK3.5 (130)	100/100 (130)	2e-90

Table S9. Viral structural proteins identified by Mass Spectrometry.

Phage	Gene	Putative protein function	Molecular Weight (KDa)	Peptide count	Sequence coverage (%)
PT9.1	ORF16	tape measure protein	96.311	8	11.60
	ORF17	distal tail protein	54.268	7	15.51
	ORF07	major capsid protein	41.978	12	40.97
NIIg9.7	ORF17	tail fiber protein	138.423	10	12.77
	ORF15	tape measure protein	95.742	20	27.58
	ORF16	distal tail protein	54.274	11	22.85
	ORF06	major capsid protein	41.846	16	63.34
PK5.1	ORF14	tape measure protein	94.690	31	33.03
	ORF16	structural protein	64.536	9	22.84
	ORF05	major capsid protein	42.008	24	69.00
	ORF04	Clp protease	27.112	5	26.33
PK3.5	ORF16	putative tail fiber protein	105.000	18	27.67
	ORF14	tape measure protein	92.728	35	38.01
	ORF15	distal tail protein	53.542	6	19.07
	ORF05	major capsid protein	41.952	17	55.25
	ORF09	putative tail component	14.216	2	16.66
PK3.6	ORF16	putative tail fiber protein	140.495	26	24.33
	ORF14	tape measure protein	91.984	25	32.22
	ORF05	major capsid protein	41.952	14	48.24

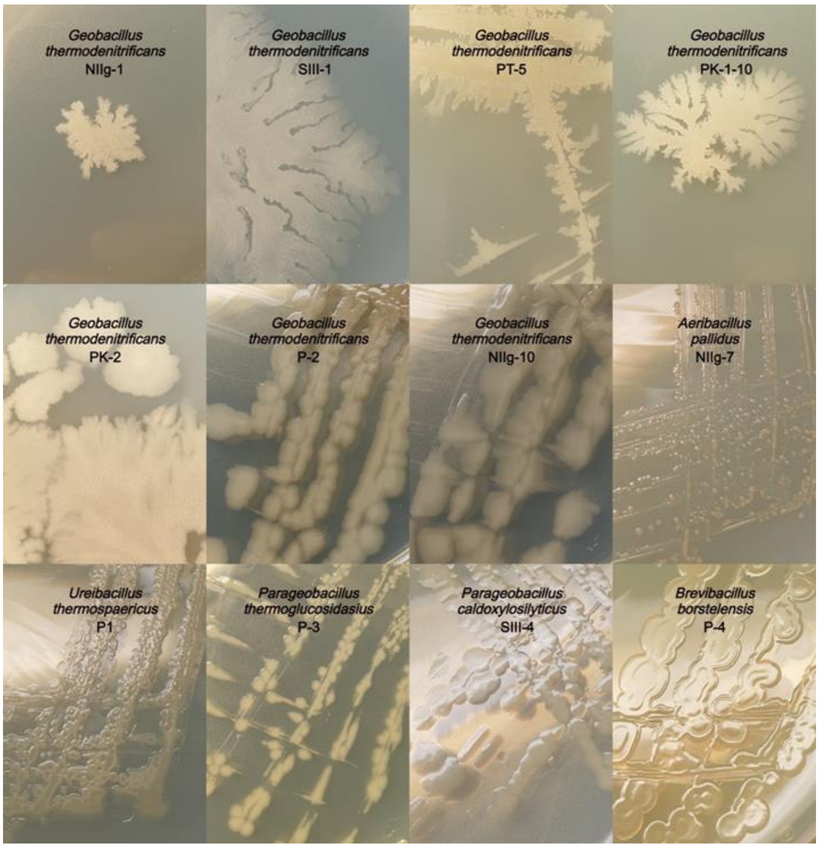

Figure S1. The morphology of colonies of isolated bacterial strains after 24 hours of incubation on LB agar medium at 55°C.

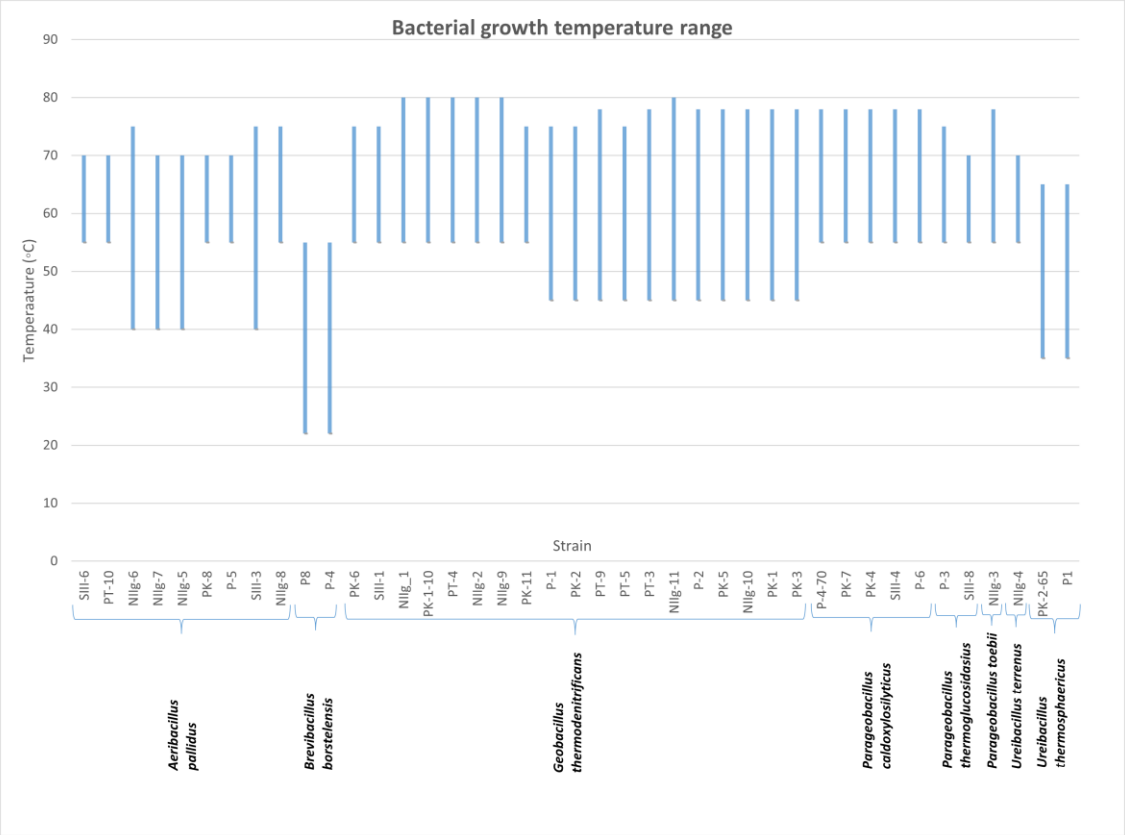

Figure S2. Bacterial growth temperature ranges. Bacterial isolates are indicated above, LB agar medium and gelan medium were used for growth experiments.

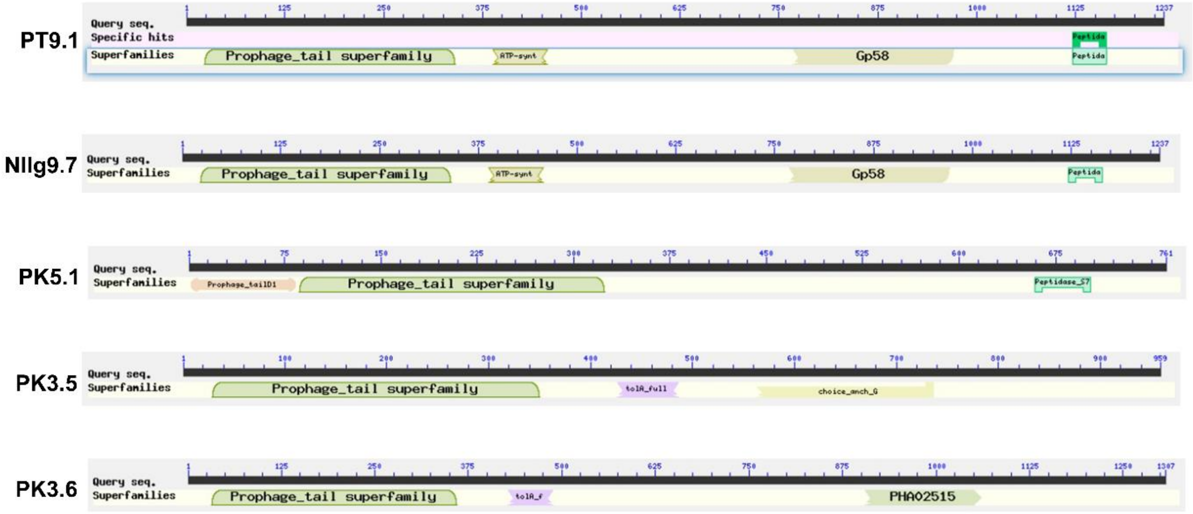

Figure S3. Comparison of conserved domains in potential depolymerase genes of thermophilic bacteriophages PT9.1, NIIg9.7, PK5.1, PK3.5, and PK3.6. Conserved domains were detected using NCBI BLASTp database.
